# Additive-Free Compatibilization of Commodity Polypropylene/Polyethylene via Partial Melting and Recrystallization

**DOI:** 10.1021/jacsau.6c00677

**Published:** 2026-05-26

**Authors:** Carmen B. Dunn, Yunjia Zhang, Peiran Wei, Jung Bin Ahn, Wenlin Zhang, Zhe Qiang

**Affiliations:** † School of Polymer Science and Engineering, University of Southern Mississippi, Hattiesburg, Mississippi 39406, United States; ‡ Department of Chemistry, 3728 Dartmouth College, Hanover, New Hampshire 03755, United States; § Soft Matter Facility, Department of Materials Science and Engineering, Texas A&M University, College Station, Texas 77845, United States

**Keywords:** Sustainability, Semicrystalline, Polyolefins, Interface Engineering

## Abstract

Mixed polyethylene (PE) and polypropylene (PP) generate roughly 250 million tons of plastic waste annually, yet their mechanical recycling still relies on additive- and reactive chemistry-based compatibilization, raising costs and complicating waste streams that can obstruct circularity at scale. Here, we report an additive-free, thermal solvent immersion annealing strategy that compatibilizes PP/PE blends post-manufacturing. Elevated temperatures partially melt crystalline regions while the solvent selectively swells amorphous domains, broadening PP/PE interfaces and promoting local chain mixing. Upon solvent removal, chain recrystallization generates entangled intercrystallite loops that bridge PP and PE domains, enabling efficient stress transfer and transforming brittle blends into tough, strain-hardening materials. We demonstrate broad applicability across PP/PE compositions and molecular identities, supported by experiments and molecular dynamics simulations that directly track loop formation and its role in stress transfer during sample deformation. This work establishes a new compatibilization pathway based on entangled intercrystallite loop formation, enabled by partial melting and recrystallization, and offers a scalable route to mechanical recycling of plastic waste via phase-specific engineering.

## Graphical abstract

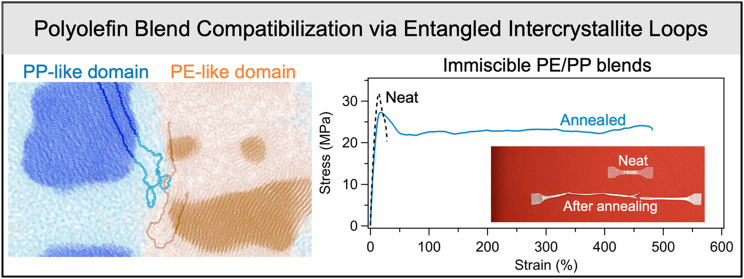

## Introduction

Semicrystalline polyolefins, such as polypropylene (PP) and polyethylene (PE), are ubiquitously used in packaging, transportation, and energy applications due to their low cost, chemical resistance, and reliable mechanical performance. However, their growing global production contributes significantly to plastic waste accumulation, posing pressing environmental and sustainability challenges.^1^ A key obstacle to recycling PP and PE stems from their similar physical properties, including comparable densities and refractive indices, which make them difficult to separate using conventional sorting processes.^2^ Consequently, PP and PE are often processed together in municipal and industrial waste streams. However, when combined, these polymers are inherently immiscible, leading to macrophase separation, poor interfacial adhesion, and severe embrittlement in recycled blends.

Recent efforts have explored both chemical and mechanical recycling strategies to address commingled PP/PE. Chemical approaches, such as catalytic cracking or polymer functionalization,^3–8^ could generate high-value products but often require high temperatures or multistep synthesis. Mechanical recycling offers a more direct path for integration into existing polymer industries, yet achieving robust performance in recycled blends often relies on the introduction of compatibilizers.^9–11^ These compatibilizers typically consist of at least two distinct components, which can selectively localize at the interfaces of immiscible polymer blends to interact with different phases, including via tie-chain formation and cocrystallization mechanisms to enhance mechanical properties.^12–16^ Notably, the efficiency of co-crystallization can be highly sensitive to processing conditions, particularly the sample cooling rate.^17^ While recent advances in compatibilizer design and synthesis improved their cost-efficacy, their incorporation can alter blend composition and complicate feedstocks, limiting opportunities for future recycling cycles.

This work introduces an additive-free annealing strategy that compatibilizes PP/PE blends across a wide range of compositions and molecular identities. Semicrystalline polyolefins provide unique dual-phase nature, in which amorphous regions are deformable and can be readily penetrated by solvents, while crystalline domains provide mechanical strength and solvent resistance. Our method combines solvent-induced swelling of amorphous regions with thermal annealing to partially melt crystalline domains. Upon drying, recrystallization of polymer chains introduces entangled intercrystallite loops that reinforce the blend interface, providing a new mechanism to significantly enhance mechanical performance without altering the overall blend composition or macroscopic morphology. We note that prior literature suggests thermal annealing alone may strengthen PP/PE interfaces without the use of additives, as evidenced primarily by improved adhesion in layered homopolymer assemblies.^18^ However, comparable demonstrations of bulk mechanical toughening in brittle, phase-separated PP/PE blends remain scarce. In contrast to conventional mechanical recycling approaches that rely on modifying resins prior to processing, our simple method compatibilizes postmanufactured polyolefin blends, making the two routes complementary and potentially well suited to work together. This work not only can enable mechanical recycling of commingled PP/PE at scale but also offers new fundamental insights for phase-specific engineering in semicrystalline polymer systems.

## Creating Entangled Loops between Distinct Polymer Crystallites

Figure 1A illustrates our thermal solvent immersion annealing strategy for compatibilizing semicrystalline PP/PE blends. In this process, solvent swelling selectively expands the amorphous regions of the polymer matrix, while the preservation of crystalline domains maintains sample structural integrity and macroscopic shape. At elevated temperatures, controlled partial melting of crystalline lamellae in conjugation with the presence of solvent broadens the interfacial zone between PP and PE phases, enabling enhanced intermolecular interactions. Upon solvent removal and recrystallization, the material recovers its original dimensions and mass, while entangled loops formed across distinct crystallites reinforce the PP/PE interface. Distinct from conventional strategies that modify resin composition prior to processing, our method enables compatibilization of post-manufactured parts, enhancing mechanical performance without changing the material chemistry or macroscopic structure. Furthermore, while this approach leverages a similar strategy to solvent-induced polymer welding of amorphous glassy polymers, it is still distinguished through the creation of unique load-bearing entangled loops between crystals.^19,20^ In our model system, blends of isotactic PP (PP) with low-density PE (LDPE) were formulated to mimic the mechanical recycling of polyolefins without effective sorting; these blends exhibit a strain at break (ε_b_) of 88  ±  14% (Table S1), attributed to poor interfacial adhesion between the immiscible PP and PE phases.

**Figure 1:**
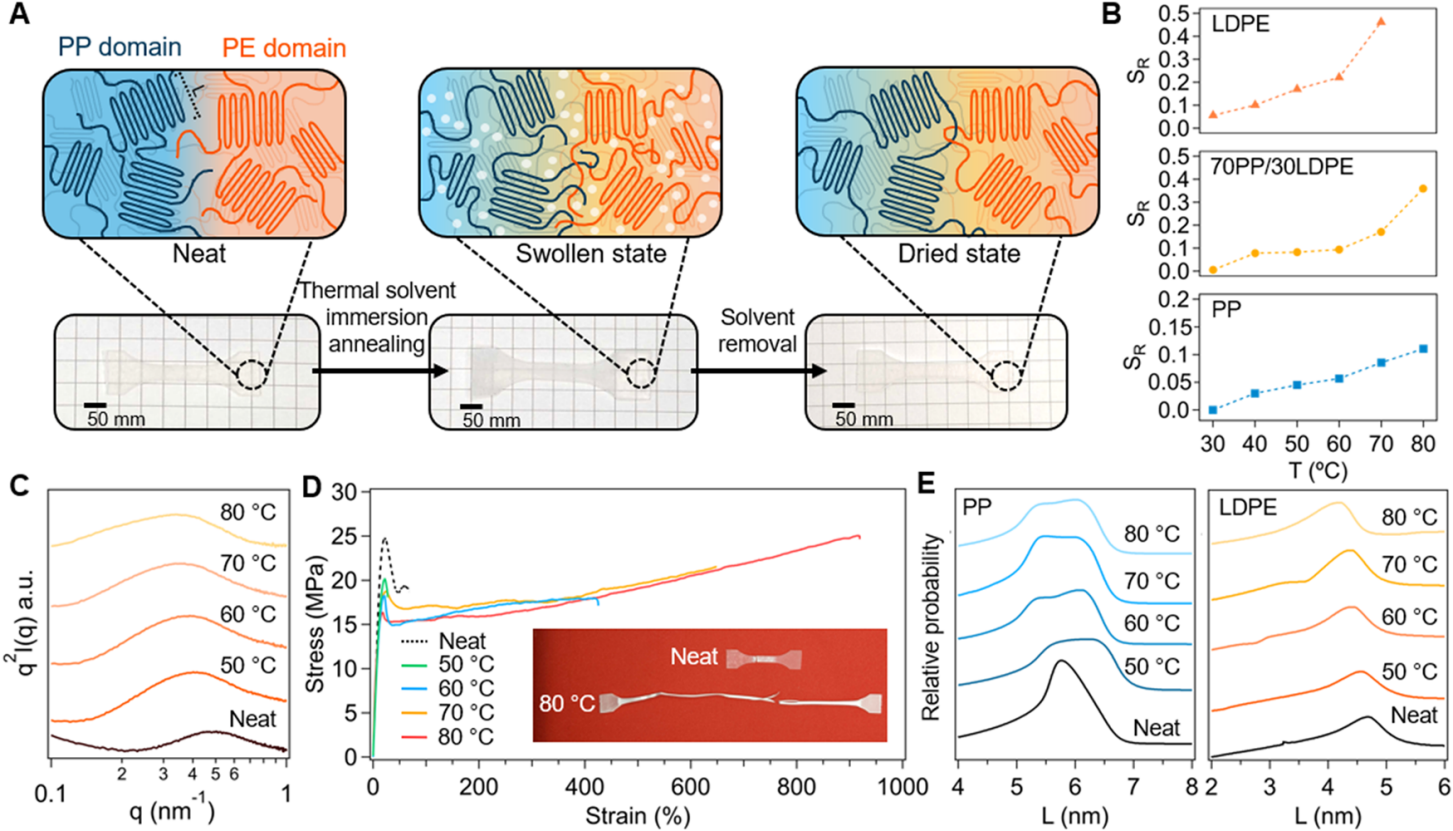
Thermal solvent immersion annealing of PP/PE blends. (A) Schematic representation of the proposed mechanism for interfacial reinforcement in PP/PE blends, highlighting solvent-induced swelling at elevated temperatures and formation of entangled intercrystallite loops across phase boundaries. (B) *S*_R_ of PP, 70PP/30LDPE and LDPE at different temperatures when immersed in xylenes after 1 h. (C) Lorentz-corrected small-angle X-ray scattering (SAXS) profiles of 70PP/30LDPE in neat state (no swelling) and during thermal solvent immersion annealing in xylenes at 50, 60, 70, and 80 °C. (D) Stress–strain curves of 70PP/30LDPE before (neat) and after thermal solvent immersion annealing in xylenes at 50, 60, 70, and 80 °C for 1 h (strain rate of 20 mm/min). (E) Lamellae thickness distributions of PP and LDPE phases in 70PP/30LDPE before (neat) and after annealing in xylenes at 50, 60, 70, and 80 °C for 1 h.

The swelling degrees (*S*_R_) of PP, PE, and 70PP/30LDPE in xylenes as a function of temperature are shown in Figure 1B, and their corresponding swelling kinetics (Figure S1) indicate that equilibrium is reached within 60 min, regardless of sample thickness. This observation is consistent with previous reports suggesting solvent penetration in semicrystalline polyolefins is facilitated by the continuous amorphous matrix.^21,22^ In general, increasing temperature leads to higher swelling ratios (*S*_R_) for all components, which is primarily due to partial melting of crystalline regions. When exposed to xylenes, LDPE exhibits *S*_R_ values of 0.17, 0.22, and 0.46 at 50 °C, 60 °C, and 70 °C, respectively. We note that at 80 °C, LDPE became dimensionally unstable in xylenes; this instability is shown in Figure S2 and is attributed to the substantial crystal melting of LDPE at this temperature, which softens the polymer samples. *S*_R_ of PP reaches 0.09 and 0.11 at 70 °C and 80 °C during our annealing process, respectively (Tables S2 and S3). For these semicrystalline polymers, swelling is governed primarily by expansion of the amorphous regions; therefore, polymers with higher crystallinity generally exhibit lower swelling because a smaller fraction of chains remains available for solvent-induced expansion. In addition, partial melting during annealing increases the amount of polymer chains in amorphous regions, which can further promote swelling. Here, we suggest that the partial melting of lamellae and mobility of freed amorphous chains near the interface enable the formation of entangled intercrystallite loops at the PP/PE interface. Since amorphous content from the PP and PE bulk must be used to facilitate these interfacial interactions, we observe a change in crystallinity after annealing (Table S4). The 70PP/30LDPE blend shows a restrained swelling behavior below 60 °C, above which the *S*_R_ increases to 0.17 and 0.34 at 70 °C and 80 °C, respectively. Furthermore, the sample mass measured before annealing, immediately after annealing in the swollen state, and after drying is summarized in Table S3, demonstrating solvent uptake content during swelling and completed solvent removal from the drying step. In addition, complete solvent removal is confirmed by Figure S3, which presents thermogravimetric analysis (TGA) of 70PP/30LDPE before and after thermal solvent immersion annealing followed by drying. In addition, high temperature gel permeation chromatography experiments of polyolefins before and after thermal solvent immersion annealing indicate the molecular weight distributions of polyolefins are retained during the process (Figure S4), confirming the process does not influence materials composition (i.e., no selective dissolution of low molecular weight species). The retention of the semicrystalline state in 70PP/30LDPE blends during thermal solvent immersion annealing in xylenes was confirmed by *in situ* small-angle X-ray scattering (SAXS). The primary scattering peak in Figure 1C, corresponding to overlapping crystalline long periods (*d*) from PP and LDPE, was preserved across the annealing temperature range from 50 °C to 80 °C, despite a shift toward lower *q* ^*^ values. The *d* of 70PP/30LDPE increased from 13.1 nm in the neat state to 15.6 nm and 19.1 nm at 50 °C and 80 °C in xylenes, attributed to partial melting of the crystalline phase and the further expansion of the amorphous phase. The *in situ* SAXS profiles for homopolymer PP and LDPE as a function of temperature during thermal solvent immersion annealing are shown in Figure S5, while *in situ* wide-angle X-ray scattering (WAXS) measurements (Figure S6) confirm the persistence of their characteristic crystalline peaks throughout our annealing process. After solvent removal, the *d* of annealed 70PP/30LDPE is close to its neat-state value, independent of annealing temperature (Figure S7). Our thermal solvent immersion annealing preserves the macroscopic phase separation morphology of PP/LDPE blends (Figure S8) with very limited alteration due to the fact that annealing is carried out while the blends remain in the semicrystalline state (both samples exhibiting an averaged domain spacing of 3–5 μm), such that portions of the polymer chains remain confined within crystalline domains. As a result, large-scale chain diffusion and macroscopic domain rearrangement are not expected during treatment. The impact of annealing method on sample degree of crystallinity (*X*_*c*_), as examined through the first heating cycles of differential scanning calorimetry (DSC), is shown in Figure S9 and Table S4. Specifically, after annealing 70PP/30LDPE for 1 h at 80 °C, the *T*_m_ of the PP phase increases from 157 to 160 °C, and its *X*_c_ increases from 0.29 to 0.33. Simultaneously, the PE *X*_c_ increases from 0.10 to 0.18, and its *T*_m_ decreases from 105 to 99 °C.

As shown in Figures 1D, S10, and Table S2, thermal solvent immersion annealing of the 70PP/30LDPE blend significantly increases the strain at break (ε_b_), reaching 596  ± 56% and 807  ±  46% after annealing in xylenes at 70 °C and 80 °C for 1 h, respectively. Correspondingly, the toughness of the samples improved from 16 ±  3 MJ/m^3^ to 105  ±  11 and 144  ±  11 MJ/m^3^. Although neat LDPE became distorted upon exposure to xylene at 80 °C, the 70PP/30LDPE blend retained its overall shape during annealing (Figure S2B). This behavior is due to the majority PP phase, which remains more dimensionally stable under these conditions and helps maintain the structural integrity of the blend even as the minority LDPE phase softens. For mechanical properties, it is found 50 °C annealing induced limited change in ε_b_ for 70PP/30LDPE (50  ±  13%), indicating a minimum temperature is required for sufficient crystallite melting to improve the mechanical property of the blends, which further suggests that the final blend properties are governed not simply by swelling, but by the annealing temperature and the structural reorganization it enables. Because thermal solvent immersion annealing has minimal effect on the toughness and ε_b_ of neat PP and LDPE homopolymers (Table S1), the observed mechanical improvements in the blends are best explained by the enhanced interfacial strength between PP and PE phases. This point is important to clarify because any polymer plasticization resulting from xylene annealing does not constitute the compatibilization mechanism. At most, it may affect stress transfer of amorphous chains, but it cannot explain the pronounced toughening of the blends. Rather, the improved mechanical performance originates from the rearrangement of interfacial structures as a result of annealing. Thus, PP/PE blends annealed at 30 °C for 1 h, which were intentionally left undried and contained approximately 3.4 wt % residual solvent, fail before 30% strain (Figure S11). Moreover, after thermal solvent immersion annealing at 70 °C, the ε_b_ of PP increases ∼50% (Figure S12), indicating that our annealing has limited effect on the overall ductility of the neat PP. Furthermore, the reduction in elastic modulus (from 254 ± 13 MPa to 214 ± 10 MPa) after thermal solvent immersion annealing at 70 °C for 1 h is attributed to the emergence of smaller PP crystallites (averaged lamellae thickness of ∼5.5 nm), as shown in Figure 1E. With respect to the reduction in yield point, we attribute this behavior to a decrease in tie-chain fraction in the bulk PP phase following thermal solvent immersion annealing. During annealing, portions of polymer chains melt and are pulled from crystalline domains into amorphous regions, allowing for chain redistribution and formation of interfacial loop structures. As a result, the number of load-bearing tie chains is reduced, which could lower the yield strength.^23^ Our control experiments on neat PP after annealing at 80 °C for 1 h also showed a decrease in yield strength (Figure S12), from 36.3 to 28.9 MPa, and a retention in extensibility and toughness, supporting that annealing-induced reorganization of crystalline structures contributes to the observed changes in elastic modules and yield strength (Table S5), yet the ε_b_ improves as a result of interfacial strengthening. Figure S13 shows the mechanical properties of 70PP/30LDPE improve as a function of annealing time (80 °C in xylenes), even in samples with larger dimensions prepared by injection molding, which ε_b_ improved from ∼192% (Neat) to ∼804% after annealing in xylenes at 80 °C for 1 h.

Lamellae thickness distributions for a blend of PP/PE before and after annealing were calculated from the first heating cycles of DSC in accordance with the Thomson-Gibbs equation (see Supporting Information). As shown in Figure 1E, in the neat state, PP and PE domains within the blend exhibit monomodal thickness distributions centered at 5.6 and 4.7 nm, respectively. After annealing at 80 °C for 1 h, PP develops a bimodal distribution centered at 5.4 and 6.2 nm, while PE lamellae thin to 4.1 nm. These results are attributed to the greater swelling of LDPE domains, enabling more extensive melting and uniform recrystallization upon solvent removal, whereas the limited swelling of PP may hinder recrystallization and result in more heterogeneous lamellar thickness distributions. Furthermore, the decrease in PE lamellar thickness, in contrast to the increase observed for the PP phase, is attributed to the different recrystallization kinetics of the two polymers during cooling and solvent removal. Because PE generally recrystallizes faster than PP, it has less opportunity for structural reorganization and full reincorporation into its original crystalline domains (more kinetically trapped), whereas the slower recrystallization of PP could allow greater reorganization prior to crystal reformation.^24^ Likewise, thermal solvent immersion annealing of PP homopolymer also leads to a bimodal lamellar thickness distribution, as derived from the first heating cycles of DSC (Figure S14).

Conventional compatibilization methods in mechanical recycling rely on additives that interact with adjacent PP or PE domains via cocrystallization and/or formation of interfacial tie chains.^13,15,25–29^ In our additive-free system, the crystallographic mismatch and chemical immiscibility between PP and PE make direct interfacial cocrystallization and chain threading highly unlikely,^9,30,31^ particularly their crystallization process remains strongly self-selective. To resolve interfacial morphology in PP/PE blends during and after thermal solvent–immersion annealing, coarse-grained molecular dynamics (CGMD) simulations were performed (simulation details described in the SI, Pages S5–S9). In the melt, the two CG polymers (polymer A: PP-like blue domain and polymer B: PE-like orange domain) spontaneously phase-separate, forming an interface where the cross-interface molecular entanglements are rare (Figure S15). The width of CG interfaces is about 5.5 times the average statistical segment length of the two polymers,^32^ resembling that of the interfaces in phase-separated PP/PE (about 3–5 nm).^13,33^ Upon cooling, the interfaces of the semicrystalline blend exhibit lower crystallinity than the bulk, as indicated by the spatial crystalline order profile (Φ*_c_*) shown in Figure 2A. We rely on the CG model because PP does not crystallize within any manageable time in atomistic simulations due to the large nucleation barrier and relatively slow segmental dynamics.^34,35^

**Figure 2:**
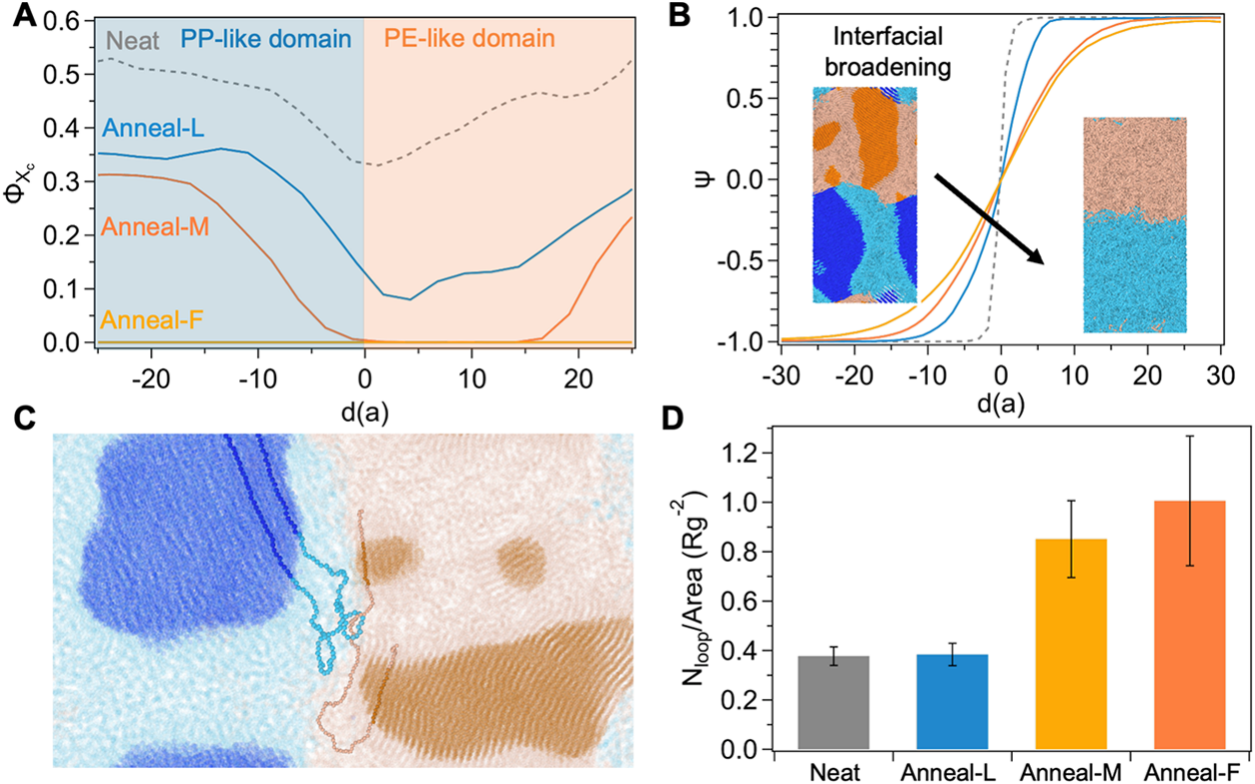
Molecular view of morphology development at the blend interface via thermal solvent immersion annealing. (A) Crystalline ordering profiles (Φ*_c_*) of two domains (left: PP-like; right: PE-like) as a function of normal distance (*d*) from the blend interface of neat sample (dashed gray) and samples during thermal solvent immersion annealing (solid). (B) Polymer composition profile (Ψ) as a function of *d* from the blend interface for the neat sample (dashed gray) and the samples during the thermal solvent immersion annealing process. ψ = (ϕ_*A*_ – ϕ_*B*_)/(ϕ_*A*_ + ϕ_*B*_), where ϕ_*i*_ represents the volume fraction of species *i*. (C) Snapshot of a simulated interface in a semicrystalline blend with highlighted entangled intercrystallite loop across the interface (darker regions represent crystallites, and lighter regions represent amorphous domains). (D) Number of entangled intercrystallite loops per blend interfacial area in neat and annealed blends after solvent removal and recrystallization. The areal density is normalized by the squared radius of gyration (*R*_g_) of polymer B. Error bars are derived from averaging over six independent simulation trajectories.

Upon introducing a good solvent to the semicrystalline blend (∼26 vol %) in our CG simulations at a temperature above the crystallization temperature, the solvent molecules can penetrate and help melt crystallites (Figure S16), which is consistent with our experimental observations. In our simulations, three samples were prepared by tuning the degree of crystal melting: Anneal-L (low), Anneal-M (moderate), and Anneal-F (full melting); Anneal-F refers to the sample rendered completely amorphous during the annealing process. Reducing the residual crystallinity of PP and PE during thermal solvent immersion annealing promotes their mixing in the amorphous phase and broadens the blend interface (Figure 2B). Experimentally, this decrease in crystallinity during thermal solvent immersion annealing is attributed to an increase in annealing temperature, which melts enough lamellae for local mixing and formation of entangled intercrystallite loops for stress transfer upon solvent removal. After solvent removal and cooling, amorphous chains undergo recrystallization while their entangled strands near the interfaces are kinetically trapped between adjacent crystallites. Particularly, when two chemically distinct, entangled polymer strands are incorporated into adjacent crystallites, they form an entangled intercrystallite loop (Figure 2C) that can transmit stress across the blend interface—a phenomenon previously observed in homopolymer systems but rarely explored in polyolefin blend interfaces.^36–38^ Specifically, while bridged molecular entanglements between crystallites of the same chemical identity have been produced in bulk through advanced characterization methods, the development of such structures (1) protruding from distinct crystallites and (2) crossing the PP/PE interface have yet to be explored as they relate to blend performance. Nonetheless, the entangled intercrystallite loops also resemble long multiblock copolymer compatibilizers, which can entangle with chain-folded loops of semicrystalline chains across the blend interfaces.^13^ As shown in Figure 2D in neat PP/PE blends, the density of such loops is low, limiting load-bearing capacity and interfacial stress transfer.^39^ The number of intercrystallite loops increases with enhanced mixing in the interfacial region and reduced crystallinity of PP and PE during annealing. Without sufficient crystal melting (Anneal-L), the moderately broadened interfaces result in negligible enhancement in intercrystallite loop formation. In a similar vein, without sufficient thermal energy to melt enough crystalline lamellae, the lack of sufficient amorphous content hinders interfacial local mixing near interfaces, and in turn results in lower loop content and reduced strain at break. Reducing the average crystallinity of the sample by approximately 42% via thermal solvent immersion annealing (Anneal-M) increases intercrystallite loop areal density by more than 2-fold—from 0.376  ±  0.038 in neat samples to 0.851  ±  0.155 after recrystallization.

## Role of Entangled Intercrystallite Loops in Stress Transfer

In PP/PE blends, maximum strain is governed primarily by stress transmission across the interface. We therefore hypothesize that increasing the density of entangled intercrystallite loops enhances elongation at break and toughness by reinforcing the interface and delaying interfacial failure. We note that the proposed mechanism bears conceptual similarity to the recently described “thread-the-needle” compatibilization mechanism, in which segments of block copolymer compatibilizers form looped conformations within selective crystalline domains, thereby contributing to interfacial reinforcement. Although that concept was developed in the context of additive-based compatibilization, it provides a useful conceptual framework for understanding how looped chain conformations generated during semicrystalline-state annealing could strengthen interfaces in the present additive-free PP/PE blends. To test this, we performed *in silico* tensile tests on neat and annealed specimens with controlled loop densities, directly correlating loop population with sample mechanical response. Representative stress-train curves for the neat and dried Anneal-M samples are shown in Figure 3A, while six-run averages for other samples are included in Figure S17. Increasing the areal density of intercrystallite entanglement loops converts the mechanical response of polyolefin blends from brittle to strain-hardening, with load-bearing loops sustaining stress transfer at large strain. Specifically, in the neat sample, the very few intercrystallite loops lead to interfacial failure at moderate (20%) strain when the bonded force exceeds typical threshold values of covalent bonds (Figure 3B), causing interfacial failure (Figure S18). In Anneal-M, the increased density of intercrystallite loops markedly improves interfacial robustness. Even at 80% tensile strain, most loops persist, and the interface remains largely intact, enabling more uniform stress distribution and the capacity of entangled loops for stress transfer (Table S6). Indeed, our simulations aim to reveal the microscopic rearrangement of polymer chains in the interfacial region at a deformation rate much higher than the experimental condition, which is inevitable in molecular simulations due to the accessible length and time scales (see SI for more details). Macroscopic deformation of the materials, such as necking, is beyond the length scale of simulations. The difference in maximum strain between the simulation and experiment is expected, as the simulation probes failure of a single interfacial region, whereas the bulk material contains numerous interfaces. In the bulk blend, these multiple interfaces can delay macroscopic fracture, consistent with mechanisms previously invoked to explain why reduced domain size enhances toughness in PP/PE blends.^15^ Nonetheless, our in silico tensile tests offer a microscopic view of the role of entangled loops in stress transmission across the blend interfaces during nonlinear mechanical deformation. Notably, the strain-hardening behavior of polymer blends is reproduced in our simulation results.

**Figure 3:**
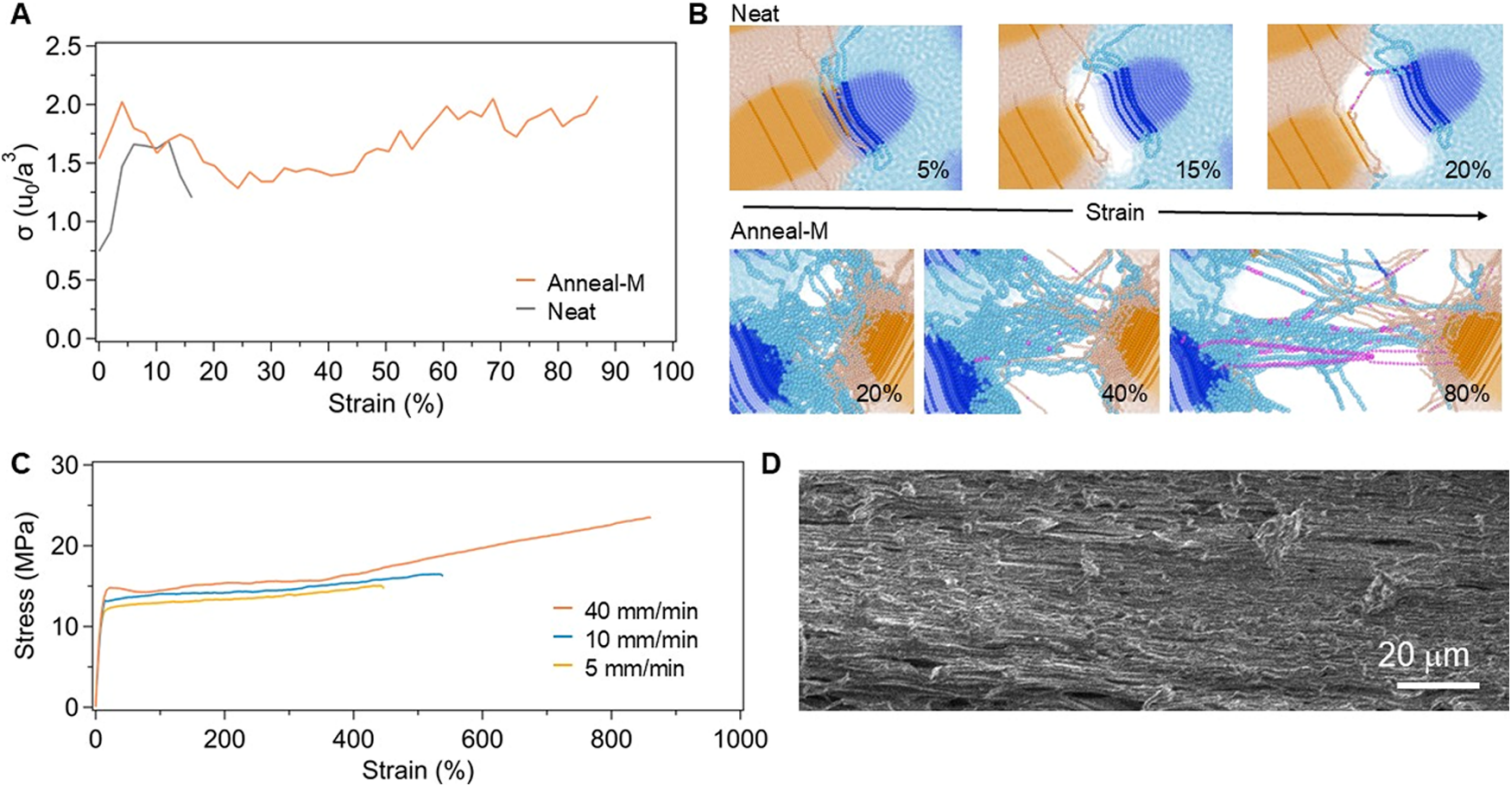
Interfacial strengthening of PP/PE blends through stress transfer. (A) Simulated stress–strain curves for neat and Anneal-M samples. *u*_0_ and *a* are the reduced energy and length units, respectively. Bonded forces in intercrystallite loops exceed the bond-breaking threshold in the neat samples at a strain of 20%. (B) Snapshots of a neat sample (top) and an Anneal-M sample (bottom) during simulated tensile tests. Bonds with bond forces exceeding the bond break threshold are labeled in pink. (C) Stress–strain curves of 70PP/30LDPE after thermal immersion annealing at 80 °C at strain rates of 40, 10, and 5 mm/min. (D) SEM image of a uniaxially strained 70PP/30LDPE sample after thermal solvent immersion annealing at 80 °C for 1 h.

Figure 3C further illustrates the impact of strain rate on the mechanical properties of the compatibilized 70PP/30LDPE blend after annealing at 80 °C in xylenes for 1 h. In general, a decrease in strain rate leads to a reduction in both ultimate strength and maximum strain. Specifically, at a strain rate of 40 mm/min, the ε_b_ was 881 ± 32%, which decreased to 489 ±  41% when the strain rate was reduced to 5 mm/min. These results are consistent with the critical role of intercrystallite loops in stress transfer. At lower strain rates, polymer chains at amorphous and interfacial regions within compatibilized blends have sufficient time to relax and reorient under loading, promoting stress dissipation rather than efficient stress transfer to the crystalline domains.^40,41^ Concurrently, the concentration of stress at blend interfaces leads to localized deformation, limiting the maximum strain prior to failure.^42^ Increasing the strain rate seems to facilitate more efficient stress transfer from the amorphous and compatibilized interfacial regions to the crystalline domains, which enhances both strength and ductility up to fracture. In contrast to the blend samples, neat PP and annealed PP (Figure S12) exhibited broadly similar strain-rate dependence in maximum elongation (all in the range of 1200%–1500%). The more pronounced strain-rate dependence observed in the annealed PP/PE blends suggests that interfacial loop formation from annealing, rather than bulk semicrystalline morphology reorganization alone, plays a central role in determining the strain-rate-dependent mechanical response of the blends. In fact, the entangled intercrystallite loops at the interfaces likely transfer stress differently from conventional bulk tie chains and may be less effective at maintaining ductility under conditions that allow greater relaxation. Figure 3D presents a scanning electron microscopy (SEM) image of compatibilized PP/LDPE after elongation up to approximately 600%, exhibiting a pronounced microfibrillar structure, characteristic of ductile deformation and efficient stress transfer through intercrystallite loops.^43^ The loops facilitate distributed stress as well as reorientation of the crystalline domains along the strain direction, which is further supported by our SAXS results. The initial isotropic scattering ring evolves into an anisotropic elliptical pattern, indicating strain-induced orientation and alignment of the crystalline lamellae structures (Figure S19).

In addition to uniaxial tensile tests, fracture mechanics of 70PP/30LDPE blends after thermal solvent immersion annealing in 80 °C xylenes for 1 h suggest entangled intercrystallite loops prevent slow crack growth and stabilize PP/PE interfaces to enable stress transfer (Figure S20). As calculated in accordance with the Thomas-Rivlin model,^44^ unannealed blends demonstrated brittle failure and a uniform fracture toughness (Γ) of ∼5000 J/m^2^, independent of ligament length (*b*). However, upon thermal solvent immersion annealing, the onset of crack propagation (λ_c_) increases from 1.07 to 1.25 (*b* = 0.75 and 1.75 mm respectively), generating an increase in both strain energy density (W­(λ*_c_*)) and Γ (Table S7). Importantly, the increase in Γ with *b* is broadly attributed to plasticity and is frequently observed in semicrystalline polyolefins and compatibilized interfaces.^45,46^ The elevated fracture toughness observed for compatibilized blends is indicative of interfacial strengthening and delay in crack onset and is consistent with bulk stress dissipation.^47,48^

## Extensions to Different Polyolefin Systems and Waste Plastic

To evaluate the broader applicability and potential limitations of our thermal solvent immersion annealing method, we extended our study to PP/PE blends with varying compositions and chemical identities (Figure 4A). For 80PP/20LDPE and 60PP/40LDPE, annealing in xylenes at 80 °C for 1 h resulted in comparable swelling behavior to 70PP/30LDPE (Figure S21) and increased the strain at break from 57  ±  15% and 31  ±  3% to 1040  ±  14% and 556  ±  38%, respectively. Correspondingly, the toughness of both blends exceeded 75 MJ/m^3^ after annealing (Figure S22). The reduced mechanical improvement at higher LDPE content can be attributed to the formation of larger LDPE domains (Figure S23). Specifically, although LDPE has a lower melting temperature and 70 °C should therefore induce sufficient crystal melting to enable loop formation, this difference may arise from the larger phase-separated domain size in 60PP/40LDPE. Larger domains likely require a greater density of interfacial loop structures to achieve effective stabilization, which in turn may require a higher annealing temperature. At LDPE loadings above 40 wt %, stable swelling during annealing at 70 °C or 80 °C was no longer achievable, likely due to excessive softening of the LDPE-majority phase. We also evaluated blends containing high-density polyethylene (HDPE), which is intrinsically brittle, is more crystalline (*X*_c_ ∼ 0.71), and exhibits a ε_b_ of ∼70 ±  7% in its neat form. After thermal solvent immersion annealing (Figure S24), the 85PP/15HDPE and 70PP/30HDPE blends reached ε_b_ values of 881 ±  32% and 392 ±  38%, respectively (Table S8 and Figure S25). The SEM images of PP/HDPE blends are shown in Figure S26, demonstrating their inherent separation. Again, complete solvent removal of different PP/PE blends after thermal solvent immersion annealing is quantified through TGA in Figure S27. Interestingly, the rate of solvent removal appears to be critical for improving extensibility of PP/HDPE blends. Interestingly, when the PE phase is more crystalline (HDPE), the efficacy of thermal solvent immersion annealing for compatibilization is limited by the solvent removal rate (Figure S28). When HDPE content exceeds 40 wt %, our annealing method no longer improves the mechanical performance of the blend, likely due to the limited degree of swelling associated with highly crystalline HDPE domain (Figure S25).

**Figure 4:**
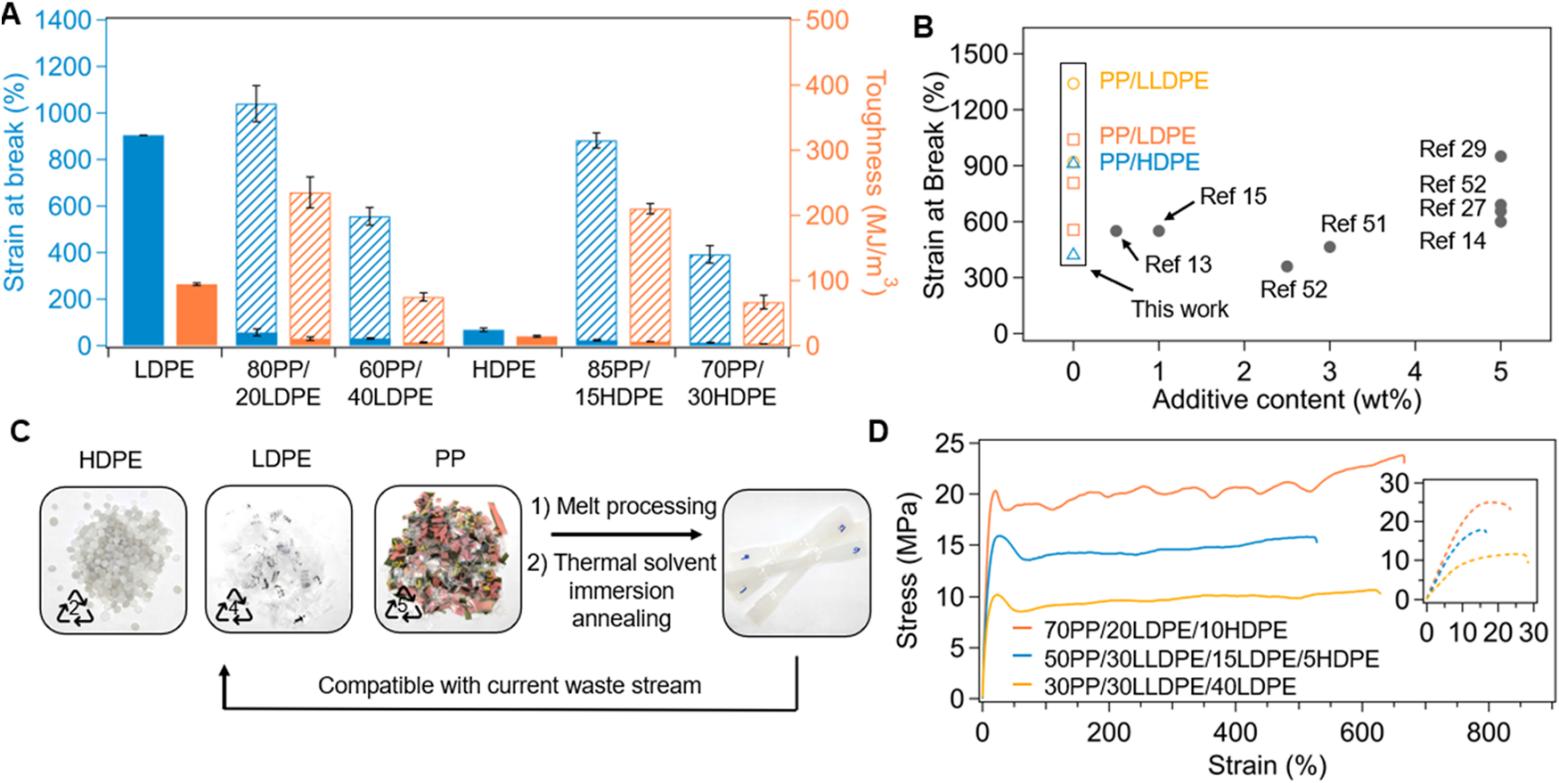
Thermal solvent immersion annealing of mixed plastic wastes. (A) ε_b_ and toughness of PP/PE blends containing LDPE and HDPE at different loadings before (solid) and after (striped) annealing. (B) Comparison of ε_b_ of PP/PE blends achieved from thermal solvent immersion annealing to other state-of-the-art blend compatibilizer technologies at different additive content levels.^13–15,27,29,51,52^ (C) Closed-loop mechanical recycling of mixed polyolefin wastes using thermal solvent immersion annealing to improve mechanical performance without the introduction of additives. (D) Stress–strain curves of mixed plastic waste with varied compositions and identities before (dashed) and after (solid) thermal solvent immersion annealing.

Interestingly, neat 70PP/30LLDPE and 30PP/70LLDPE blends exhibit ductile behavior even before annealing (Figure S29). Upon annealing in xylenes at 80 °C for 1 h, the ε_b_ of these blends is further enhanced, reaching 836 ±  50% and 1275 ±  38% for 70PP/30LLDPE and 30PP/70LLDPE, respectively, indicating that the method remains effective even in systems with favored compatibility. *In situ* SAXS results confirm that both LLDPE and HDPE remain semicrystalline during the annealing process (Figure S30), and DSC first heating cycles illustrate the retention of two characteristic crystalline features corresponding to respective PP and PE domains (Figure S31 and Table S9). Interestingly, there is a more notable shift in the *d* of LLDPE (from 19.2 to 28.7 nm) than HDPE (from 22.4 to 24.3 nm) during thermal solvent immersion annealing at 50 and 80 °C respectively (Figure S30), which is attributed to the lower degree of crystallinity and greater amorphous content of LLDPE, enabling a higher degree of swelling. Across different blend systems, we expect the extent of crystal melting of PE and PP during immersion in xylene to be broadly similar at a given annealing temperature, provided the materials have the same identity (e.g., tacticity, linear or branched chain structure, molecular weight, etc.). As shown in Figure 4B, our additive-free strategy achieves mechanical performance in commingled PP/PE blends that is comparable to—or exceeds—that of state-of-the-art compatibilization approaches based on mechanical recycling with the introduction of advanced blend compatibilizers at different content levels, including both maximum strain and the elastic moduli of PP/PE blends (Table S10).^13–15,17,27,29,49–58^ As our method targets post-manufacturing improvement and previous approaches focus on resin modification, we believe thermal solvent immersion annealing could function as a complementary strategy to existing compatibilizer systems, providing a universal and modular approach for addressing diverse waste streams.

Figure 4C shows our method applied to real postconsumer polyolefin waste streams, which often feature complex multiphase compositions due to various grades of PE and PP. We collected mixed plastic waste from sources including cups, bottles, textiles, bags, and packaging (Figure S32). These materials were ground, compounded, and processed into tensile bar specimens, followed by thermal solvent immersion annealing. A gratifying feature of our method is the absence of additives in the compatibilized plastic waste, allowing for continued downstream recycling without introducing new components. To demonstrate our method’s capability for addressing polyolefin blends after multiple recycling cycles, Figure S33 shows thermal solvent immersion annealing of the 70PP/30LDPE blend following repeated melt reprocessing yielded comparable mechanical performance across all samples.

To more accurately model real feedstocks, more complicated blend compositions were prepared with combinations of postconsumer PP, LLDPE, LDPE, and HDPE wastes (Figure 4D). In neat form, these blends were all very brittle, but thermal solvent immersion annealing was effective in swelling these materials (Figure S34) and enhancing their mechanical performance. Specifically, a blend of 70PP/20LDPE/10HDPE demonstrated a significantly elevated ε_b_ of 660% after annealing in xylenes at 80 °C for 1 h, while the neat blend was extensible to only 25% strain before fracture. Likewise, we have demonstrated a modular approach for different ternary and quaternary waste streams, where our thermal solvent immersion annealing of 50PP/30LLDPE/15LDPE/5HDPE (60 °C for 1 h) and 30PP/30LLDPE/40LDPE (70 °C for 1 h) in xylenes enabled sample toughness to surpass 60 MJ/m^3^ in both systems, which is over a 30-times increase compared to the neat blends. These results show that our method is effective for multicomponent polyolefin waste streams and adaptable to a wide range of compositions. Although catalyst origin may influence PP microstructure through differences in molecular weight distribution and stereoregularity,^59^ the brittle-to-ductile transition induced by thermal solvent immersion annealing was observed across all PP/PE systems examined here, confirming its robust application.

## Conclusion and Outlook

Mechanical recycling of mixed polyolefins remains a significant challenge at scale. The difficulty of compatibilizing polyolefin blends stems from the wide range of processing conditions, molecular weights, chain architectures, and blend compositions involved, which prevents the application of a “one-size-fits-all” solution. Existing additive-based compatibilization strategies, including both reactive and nonreactive systems, have led to important advances in the design of polyolefin blends and can be highly effective. At the same time, these approaches often rely on tailored copolymer synthesis, careful control over composition- and processing-dependent interfacial morphology of PP/PE blends, and the introduction of additional components that may complicate downstream recyclability. In this context, our work offers a simple and additive-free alternative based on mild heating in a recoverable xylene environment. Rather than introducing a separate compatibilizing component, this approach leverages the structural duality of semicrystalline polyolefins to promote compatibilization through the formation of entangled intercrystallite loops across polymer domains. These molecular bridges strengthen the interface and lead to substantial improvements in mechanical performance.

More broadly and critically, we view our annealing strategy not necessarily as a replacement for additive-based compatibilization, but as a complementary framework that may expand the available toolbox for polyolefin blend recycling design. Because no foreign compatibilizer is introduced, this method may offer advantages in terms of simplified material identity and downstream recyclability. At the same time, the mechanistic insights developed here may also inform future hybrid strategies in which thermal solvent immersion annealing is combined with reduced compatibilizer loadings to further enhance performance. Importantly, the annealed materials remain fully recyclable after thermal solvent immersion annealing, enabling seamless reintegration into polyolefin waste streams without disrupting downstream processing. The solvent is readily recoverable, further improving sustainability and industrial feasibility of our method, and bulk thermal solvent immersion annealing as post-manufacturing allows for the accommodation of several parts and geometries with opportunities to scale up for larger industrial applications. We recognize that the current xylene-based solvent system has limitations, particularly with respect to potential toxicity and low volatility (long time drying is required). Accordingly, future work will focus on identifying alternative and more sustainable solvent candidates that may provide a more favorable balance of performance, safety, and sustainability.

Nevertheless, recent developments in compatibilizer technology have improved the trajectory of plastics circularity to address mixed plastics postconsumer, and semicrystalline state engineering of polyolefins through partial melting and recrystallization could be complementary to these approaches. Together, thermal solvent immersion annealing represents a practical mechanical recycling strategy for mixed polyolefin waste, opening new pathways for circular plastics processing at scale.

## Supplementary Material



## Notes

Safety; materials; sample preparation; thermal solvent immersion annealing protocol; characterization methods; molecular dynamics simulations; simulated tensile tests; dimensional swelling kinetics and DSC results from 70PP/30LDPE tensile bars of different dimensions; images of LDPE and 70PP/30LDPE before and after thermal solvent immersion annealing; thermogravimetric analysis (TGA) of 70PP/30LDPE before and after thermal solvent immersion annealing; high temperature gel permeation chromatography of polyolefins before and after thermal solvent immersion annealing; *in situ* SAXS as a function of PP and LDPE annealing temperature; *in situ* WAXS of PP, LDPE and 70PP/30LDPE as a function of annealing temperature; SAXS of 70PP/30LDPE before and after annealing at different temperatures; SEM of 70PP/30LDPE before and after annealing; DSC of 70PP/30LDPE before and after annealing at different temperatures; images of tensile replicates of 70PP/30LDPE after thermal solvent immersion annealing; stress–strain curves of 70PP/30LDPE containing xylenes; stress–strain curves of PP at different strain rates before and after thermal solvent immersion annealing; stress–strain curves of 70PP/30 LDPE as a function of annealing time; DSC of PP and LDPE before and after annealing; normalized entanglements near a noncompatibilized interface; simulated thermal solvent immersion annealing; simulated stress–strain curves of neat and annealed blend interfaces; snapshots of neat samples during simulated tensile tests; 2D SAXS patterns of 70PP/30LDPE with applied strain; fracture mechanics of 70PP/30LDPE; swelling ratio of PP/LDPE blends at different temperatures; stress–strain curves of PP/LDPE blends after annealing at different temperatures; SEM of 60PP/40LDPE after annealing; swelling ratio of 70PP/30HDPE as a function of time and temperature; stress–strain curves of PP/HDPE blends before and after annealing; SEM images of PP/HDPE blends after annealing; TGA of PP/PE blends; stress–strain curves of PP/PE blends with different drying conditions; stress–strain curves of PP/LLDPE blends before and after annealing; *in situ* SAXS of LLDPE and HDPE as a function of annealing temperature; DSC of PP/LLDPE blends as a function of annealing temperature; image of post-consumer polyolefin waste used for mechanical recycling in the study; stress–strain curves of 70PP/30LDPE after multiple recycling and annealing cycles; swelling ratios of ternary and quaternary PP/PE blends at different annealing temperatures; mechanical and crystalline properties of PP, LDPE, LLDPE, HDPE, and 70PP/30 LDPE before and after annealing at different temperatures; average strain at break of neat and annealed simulated interfaces; mechanical and crystalline properties of PP/PE blends before and after thermal solvent immersion annealing; strain at break and modulus data of state-of the art compatibilized PP/PE blends; supplemental references 1–24 . The following files are available free of charge (PDF)
