# Supplementary material for: Additive-Free Compatibilization of Commodity Polypropylene/Polyethylene via Partial Melting and Recrystallization
Source: JACS Au. 2026 May 26;6(7):4176–86. doi: 10.1021/jacsau.6c00677 (PMC13417184; doi:10.1021/jacsau.6c00677)
Supplement: Supplementary file 1 [file au6c00677_si_001.pdf]

Supporting Information For:

Additive-free compatibilization of commodity polypropylene/polyethylene via partial melting and recrystallization

Carmen B. Dunn[1], Yunjia Zhang[2], Peiran Wei[3], Jung Bin Ahn[3], Wenlin Zhang[2], Zhe Qiang[1*]*

[1]School of Polymer Science and Engineering, University of Southern Mississippi, Hattiesburg, MS, 39406 United States.

[2]Department of Chemistry, Dartmouth College, Hanover, NH, 03755 United States

[3]Soft Matter Facility, Department of Materials Science and Engineering, Texas A&M University, College Station, TX, 77845 United States

*Corresponding authors. Zhe Qiang and Wenlin Zhang

Experimental Methods

Safety

No unexpected or unusually high safety hazards were encountered.

Materials

Polypropylene (PP, extrusion-grade, weight-averaged molecular weight: ~635,000 g/mol, Đ: ~4.8) was obtained from Muehlstein. Low-density Polyethylene (LDPE weight-averaged molecular weight: ~1,769,000 g/mol, Đ: ~7.9) was obtained from Chevron Phillips. High-Density Polyethylene (HDPE, weight-averaged molecular weight: ~908,000 g/mol, Đ: ~6.5) was obtained from Nova Chemical. Linear Low-Density Polyethylene (LLDPE, weight-averaged molecular weight: ~1,813,000 g/mol, Đ: ~6.7) was obtained from ASTM International. Post-consumer waste plastic (including PP, HDPE, LDPE, and LLDPE) was sourced from community recycling bins designated for recyclable household waste."

Sample preparation

All formulations were made in a Thermo Scientific Process 11 corotating twin screw extruder in 100 g batches using a process temperature of 190 ˚C and a screw speed of 250 rpm. Blends are named with the convention of (weight percent PP) PP/(weight percent PE) PE type. For an example blend composition of 30 wt.% LDPE (70PP/30LDPE), PP (70 g) and LDPE (30 g) were pre-mixed and introduced into the extruder at a 16 rpm feed speed and were processed at a residence time of 5 min. Additionally, for preparing polyolefin blends from post-consumer wastes, plastic waste was ground and cut into small pieces prior to compounding.

Pelletized blends were distributed into rectangular aluminum molds and compression molded with a Carver press at ~34.5 MPa for 5 min at 190 ˚C before cooling between two metal plates until samples reached room temperature (approximate cooling rate of 20 ˚C/min). Following ASTM D638 standards, samples were then punch-pressed into Type V tensile bars (width (w) = 3.25 mm, thickness (t) = 0.75 mm, length (l) = 12.25 mm) for further study. This sample size was used throughout this work as a model system for preparing polyolefin films. For samples with larger dimensions, ASTM D638 Type I tensile bars (w = 13 mm, t = 3.2 mm, l = 50 mm) were prepared via injection molding. Specifically, pelletized blends were injection molded using a BOY22A PRO. A shot size of 43 mm was used with an injection pressure of 1.8 MPa, an injection velocity of 110 mm/s, and a screw speed of 400 rpm. The barrel temperature was set to increase from 220 ˚C at the back of the barrel to 240 ˚C at the nozzle with a feed throat temperature of 60 ˚C. Injection time was held at 15 s, and cooling time was held at 12 s (approximate cooling rate of 200 ˚C/min). To mimic multiple recycling events of PP/PE waste, reprocessing of fractured 70PP/30LDPE samples was carried out with a Carver press at ~34.5 MPa for 5 min at 190 ˚C followed by cooling between two metal plates until samples reached room temperature (approximate cooling rate of 10 ˚C/min); this process was followed for two and three cycles.

Thermal solvent immersion annealing

For a typical immersion annealing protocol, tensile bar specimens (up to 20 Type V bars) were immersed in a beaker containing xylenes (~200 mL) at various temperatures ranging from 30%˚C to 90%˚C, for durations spanning 5 min to 4 hours. The solvent was continuously stirred at 80%rpm throughout the annealing process to ensure uniform thermal and solvent exposure to the sample. At the conclusion of the designated annealing time, samples were promptly removed from the solvent and transferred to a hood at room temperature, where they were allowed to dry under vacuum at room temperature for at least 24 hours. Both sample mass and dimension were recovered to its original state after drying. Samples were subsequently dried at 60 ˚C under vacuum for an additional 48 hours (for a total standardized drying time of 72 hours). The specific annealing conditions applied to different sample sets are summarized as follows:

For all PP/LDPE model systems, annealing was conducted at temperatures ranging from 30%˚C to 90%˚C, with a fixed annealing duration of 1 hour unless otherwise specified. For the specific case of 80%˚C, samples were annealed for varying durations of 5, 15, 30, 45, and 90 min, as well as 1 hour, to investigate the effect of annealing time on material properties. An equilibrium swelling time was taken to be 1 hour for all samples.

For all PP/HDPE systems, annealing was conducted at 90 ˚C in xylenes with a fixed duration of 4 hour after which, samples were removed from solvent and then dried under vacuum for at least 24 hour before mechanical properties testing. For all PP/LLDPE systems, annealing was conducted at 70 ˚C in xylenes with a fixed duration of 1 hour after which, samples were removed from solvent and dried under vacuum for at least 24 hours before mechanical properties testing.

To probe the impact of solvent removal rate on the performance of 70PP/30LDPE and 70PP/30HDPE blends after thermal solvent immersion annealing, two conditions were used. After annealing 70PP/30LDPE (80 ^{0}C/1 hour) or 70PP/30HDPE (90 ^{0}C/4 hours), samples were subjected to rapid or slow solvent removal. Samples with rapid solvent removal were removed from the solvent bath and dried according to the protocol described above. Samples with slow solvent removal were left in the solvent bath, and the heating was turned off to allow for slow cooling in the presence of solvent. Once the solvent bath reached room temperature (after ~ 2 hours), samples were removed and dried under vacuum as described above.

For mixed plastic waste systems, a fixed annealing duration of 1 hour was employed. In the case of the 70PP/20LDPE/10HDPE blend, materials were annealed in xylenes at 80 ˚C. Samples of 50PP/30LLDPE/15LDPE/5HDPE were annealed in xylenes at 60 ˚C, and the 30PP/30LLDPE/40LDPE was annealed in xylene at 70 ˚C. The drying conditions for the ternary and quaternary blends were held constant; samples were removed from xylenes and dried under vacuum for at least 24 h before mechanical properties testing.

Characterization

The mass of polyolefin samples before (m_i) and immediately following (m_s) thermal solvent immersion annealing was determined using a digital analytical balance, and their physical dimensions were obtained using a digital caliper. Additional mass measurements were taken after drying (m_f) to monitor solvent removal (**Table S3**).

The swelling ratio was defined based on dimensional change as:

$$\text{Swelling ratio } (S_R) = \frac{L_t - L_0}{L_0}$$

where L_0 and L_t are the sample dimensions (e.g., thickness, length, or volume) before and after annealing, respectively. Swelling ratio was used to quantify the degree of dimensional expansion due to solvent uptake (**Table S3**).

The melting and crystallization temperatures (T_m and T_c) of polyolefins were determined using differential scanning calorimetry (DSC) with a TA Instruments Discovery DSC250. Tzero pans and lids from TA Instruments were used, and a heat-cool-heat cycle was employed with a temperature profile of 25 to 200 ˚C with a ramp rate of 10 ˚C/min. For samples after thermal solvent immersion annealing, the first heating cycle was used to characterize the post-annealing crystalline structure of the polyolefins. Distributions of crystalline lamellae thicknesses can be derived from DSC melting isotherms taken from the first heating cycle using a modified form of the Thomson–Gibbs equation as follows:

$$\frac{1}{M}\frac{dM}{dL} = \frac{dE}{dT}\frac{\left(T_m^0 - T_m\right)^2 \rho_c}{2\sigma_e T_m}$$

where σ_e is the surface energy of the basal surface of a lamellae (PP = 62.3×10^{-7} J/cm^2 , PE = 60.9×10^{-7} J/cm^2), $T_m{}^o$ is the equilibrium melting temperature (PP = 481 K, PE = 415 K), M is the mass of the crystalline phase, dE/dT is the energy needed to melt dM of the crystalline phase, ρ_c is the density of the crystal phase (1 g/cm^3), and $\Delta H_f{}^o$ is the reference enthalpy of fusion for the crystalline phase (PP = 207 J/g, PE = 293 J/g). Furthermore, the relative degree of crystallinity (X_c) was calculated from the following relationship:

$$X_c = \left(\frac{\Delta H_{fi}}{\Delta H_{fi}^0}\right) \div w_i$$

where $\Delta H_{f\,i}$ is the measured enthalpy from integration of the melting endotherm of species i, $\Delta H^0_{f\,i}$ is the reference enthalpy of fusion for the crystallite of species i, and w_i is the weight fraction of species i, in the blend.

Thermogravimetric analysis (TGA) of PP/PE blends before and after thermal solvent immersion annealing was carried out on a TA Instruments Discovery 550 Thermogravimetric Analyzer with platinum high temperature pans. A ramp rate of 30 ˚C/min was employed from room temperature to 600 ˚C. High-temperature size exclusion chromatography (HT-SEC) was performed on an HLC-8321GPC/HT system (Tosoh Bioscience, USA) equipped with a refractive index detector and two TSKgel GMHHR-H(20) HT2 columns. The measurements were conducted at 160 ˚C using 1,2,4-trichlorobenzene as the mobile phase at a flow rate of 0.5 mL min^{-1}. Polymer samples were prepared at a concentration of 4 mg mL^{-1} and heated at 160 ˚C for 2 h prior to analysis to ensure complete dissolution. Butylated hydroxytoluene (BHT, 0.1 wt%) was added as a stabilizer to suppress polymer degradation at elevated temperature. Molecular weights were reported relative to polystyrene standards.

Both small-angle and wide-angle X-ray scattering (SAXS and WAXS) were performed on neat PP,LDPE, HDPE, LLDPE and 30PP/70LDPE using a Xeuss 3.0 system (Xenocs, Grenoble, France) equipped with a GeniX beam delivery system, providing monochromatic Cu and Kα radiation. Scattering patterns were collected using a Dectris Eiger 2R 1M-pixel 2D detector and reduced to integrated 1D profiles. For the in situ thermal solvent immersion annealing experiment, each sample was loaded into a 1.5 mm capillary filled with xylene and mounted on a temperature-controlled Linkam stage. Measurements of PP, LDPE, and the 70PP/30LDPE blend were taken at 30, 40, 50, 60, 70, and 80 ˚C. For HDPE and LLDPE, SAXS and WAXS were performed at the 5-ID-D beamline at the Advanced Photon Source within Argonne National Laboratory. A q-range of approximately 0.034 nm^{-1} to 8.8 nm^{-1} was investigated using an x-ray energy of 9 keV. The 2D images were azimuthally averaged to provide 1-dimensional data using programs developed at 5-ID-D. All long periods were calculated using the relationship: $d = 2\pi/q$.

Tensile tests of Type V bars were performed on a Mark-10 EasyMESUR motorized test stand with wedge grips and a 250 N load cell, based on ASTM 638, where a strain rate of 20 mm/min based on crosshead displacement was used to compare the performance of samples before and after annealing. Additional tensile tests were conducted at strain rates of 5, 10, and 40 mm/min to study the stress-transfer mechanism of entangled intercrystallite loops. Each sample condition was tested with five tensile bars and averaged; standard error was calculated and reported for each sample set. Data analysis was performed on Igor Pro 9 where the toughness was determined through integration of the stress-strain curves for each tensile bar, and the maximum strain at break was determined as the strain at the point of sample failure. The elastic modulus was calculated from the slope of the linear region of each tensile curve prior to plastic deformation. Tensile tests of Type I bars were performed on an MTS Insight standard length electromechanical tester equipped with wedge grips and a 10 kN load cell. An extension rate of 50 mm/min by crosshead

displacement was employed, based on ASTM 638. Data analyses for Type I bars followed the same protocol as Type V bars. Scanning electron microscopy (SEM) images were taken with a SNE-Alpha Tabletop SEM (NanoImages). The samples were first cryo-fractured by soaking samples in liquid nitrogen for 10 min, then fractured in liquid nitrogen with pliers. Prior to imaging, the fractured cross-section was sputter-coated with MCM-100 ion sputter coater (SEC Co., Ltd.) to achieve a 5 nm thick gold layer to enhance conductivity. The microscope is operated at an acceleration voltage of 5 kV.

For fracture mechanics of 70PP/30LDPE blends, Type V tensile bars were used (fabricated the same as above via compression molding). A single-edge notched geometry was employed, where bars were notched with a razor blade under magnification. Notch depths of 1.50, 2.00, and 2.50 mm (corresponding to ligament lengths, b, of 1.75, 1.25, and 0.75 mm, respectively) were used with five replicates each for 70PP/30LDPE blends before and after thermal solvent immersion annealing (total of 30 samples). All tests were performed at 5 mm/min (crosshead displacement) using a Mark-10 EasyMESUR motorized test stand with wedge grips and a 250 N load cell. The critical extension at which crack propagation begins (λ_c) was defined for each sample as the first drop in observed load in the load-displacement curve. The strain energy density ($W(\lambda_c)$) was calculated as the area under the stress-displacement curves from $\lambda = 0$ to $\lambda = \lambda_c$ for unnotched specimens. The fracture energy (Γ) was then calculated for each sample with the following relationship:

$$\Gamma = W(\lambda_c) \cdot h_0$$

where h_0 is the corresponding sample length.

Molecular dynamics simulations

Coarse-grained (CG) simulations were performed using the GROMACS simulation package. Our CG model has been used to study crystallization near the interfaces in phase-separated polymer blends.[1] In these simulations, each polymer chain consisted of N = 200 coarse-grained beads of type A or B, connected by harmonic springs with a stretching potential:

$$U_{\text{bond}} = \frac{1}{2} k_0 \left(l - l_{ij}\right)^2$$

where $k_0 = 1270\, u/a^2$ is the spring constant, and l_{ij} denotes the equilibrium bond length between beads of type i and j. Reduced units a and u were used for length and energy, respectively. Thus, the reduced time $\tau = \sqrt{m_b a^2/u}$, where m_b is the bead mass, which is the same for A and B beads. The time step for the molecular dynamics simulations was $\Delta t = 5 \times 10^{-3}\tau$. To prevent co-crystallization, the size of B beads was reduced to 75% of that of A beads, a ratio inspired by the lattice dimensions of polyethylene and isotactic polypropylene crystals.[2,3] The bond lengths were

accordingly scaled the in homopolymers A and B and the block copolymers as: $l_{AA} = l_{BB} = 0.75\ a, l_{AB} = 0.875\ a$.

The non-bonded interactions were described using a truncated and shifted Lennard-Jones (LJ) potential:

$$U_{ij}(r) = 4\epsilon_{ij}\mathrm{u}\left[\left(\frac{\sigma_{ij}}{r}\right)^{12} - \left(\frac{\sigma_{ij}}{r}\right)^{6}\right] - U_{ij}(r_c)\ (r < r_c)$$

where $r_c = 4.725\ a$ is the cutoff distance, and the potential is shifted by

$$U_{ij}(r_c) = 4\epsilon_{ij}\mathrm{u}\left[\left(\frac{\sigma_{ij}}{r_c}\right)^{12} - \left(\frac{\sigma_{ij}}{r_c}\right)^{6}\right]$$

The non-bonded interactions were set to be 0 if $\mathrm{r} > r_c$. We set $\sigma_{ij} = 1.89\ l_{ij}$ to enhance lamellar crystal formation. The rather large bead diameter introduced bending stiffness through 1,3-repulsive interactions, promoting local alignment of bonded beads. Unlike CG-chains with their stiffness controlled by harmonic bending potentials, our CG polymers can make tight folds into crystalline lamellae, which mimics the realistic semicrystalline morphologies in polyolefins.

To control polymer miscibility, we set $\epsilon_{AA} = \epsilon_{BB} = 1$ and tuned $\epsilon_{AB} = 0.99$ to achieve weak immiscibility between polymers A and B. To prepare phase-separated blends with planar interfaces, we first confined a melt of 150 A chains or 300 B chains between two flat and impenetrable walls perpendicular to $\hat{z}$. The periodic boundary condition was applied in the $\hat{x}$ and $\hat{y}$ directions. We then constructed a binary blend with sharp interfaces by gluing the A and B slabs together, removing the walls, and reinstating periodic boundary conditions in $\hat{z}$. The system was equilibrated at $\mathrm{T} = 3\ \mathrm{u}/k_B$ for $0.114\ \mathrm{M\tau}$, allowing interdiffusion of the two species across the interface. The pressures along $\hat{x}$ and $\hat{y}$ were maintained at zero using the Parrinello–Rahman barostat. During equilibration, the initially sharp interfaces broadened and eventually stabilized. The equilibrated interfacial width was about 6.5 times the statistical segment length of polymer B. For conceptual references, the interfacial width between head-to-head polypropylene (hhPP) and polyethylene (PE) at 450 K is about 7 nm and thus 12 times the statistical segment length of PE $b_{PE} \approx 0.59$ nm.[4,5] The interfaces between phase-separated polyolefins are expected to be about 3-5 nm,[6] which are about 5 to 8 times b_{PE}.

The NPT simulation (constant number of particles, pressure, and temperature molecular dynamics) for the equilibrated blend was further extended for an additional $0.6845\ \mathrm{M\tau}$ and 6 independent configurations were extracted, which were separated by more than twice the conformational relaxation time. The phase-separated blends were then quenched to $\mathrm{T} = 2.33\ \mathrm{u}/k_B$ and crystallized for $1.597\ \mathrm{M\tau}$. Unless otherwise stated, all the measurements were averaged over 6 crystallization trajectories for each system.

To identify crystalline atoms in simulations, we calculated the local nematic order tensor for a given atom:

$$Q_{ij} = \frac{1}{n}\sum_{k=1}^{n}\left(t_i^k t_j^k - \frac{1}{3}\delta_{ij}\right)$$

where n is the number of same-type atoms within a $4.45\ a$ cutoff from the reference atom, and t^k is the unit bond vector of neighbor k, and indices $i,j = (x,y,z)$. The scalar order parameter is computed as $S = 1.5\lambda$, where λ is the largest eigenvalue of Q. The cutoff of $4.45\ a$ corresponds to the average location of the second peaks in the radial distribution functions of the two polymers. Atoms with $S > 0.8$ were classified as crystalline.

Entanglement kinks were identified using the Z1+ program.[7] Following our previously reported procedure,[1] we computed the normalized entanglement density within the amorphous region, $\tilde{\rho_e}$ with respect to the distance to the interface. Entanglement kinks connecting polymer A and polymer B were classified as interspecies entanglements, and their corresponding normalized density $\tilde{\rho_e^i}$ was similarly evaluated with respect to interfacial distance. The spatial profiles of $\tilde{\rho_e}$ and $\tilde{\rho_e^i}$ are shown in **Figure S15**. After identifying the crystalline atoms, we froze four different fractions of crystalline atoms in our simulations. In the highest frozen ratio sample, all crystalline atoms were frozen, whereas in the lowest frozen ratio sample, no atoms were constrained (corresponding to Anneal-F). For the two intermediate samples (Anneal-L and Anneal-M), subsets of crystalline atoms were selected based on their distance from the center of mass of crystalline domains. The selection range was chosen such that approximately 20% and 30% of the total atoms were frozen, corresponding to the sample Anneal-M and Anneal-L, respectively.

Subsequently, solvent beads (S) corresponding to 15% of the total number of A and B beads were introduced into the system via random insertion. The interaction strengths were set to $\epsilon_{AS} = 1.5$, $\epsilon_{BS} = 1.6$ and $\epsilon_{SS} = 1$. To facilitate solvent penetration and swell the systems, we first used a smaller solvent bead size ($\sigma_{SS} = 1.134\ a, \sigma_{SA} = 1.512\ a,$ and $\sigma_{SB} = 1.276\ a$) and equilibrated the system at $T = 2.67\ u/k_B$ for $22.8\ k\tau$. We then increased the solvent bead size to $\sigma_{SS} = 1.654\ a, \sigma_{SA} = 1.677\ a$, and $\sigma_{SB} = 1.630\ a$ followed by an additional equilibration for $0.114\ M\tau$ to achieve complete interfacial solvation. During this equilibration period, the selected crystalline atoms were restrained to their original positions using a harmonic potential with a force constant $k_r = 127\ u/a^2$. The final volume fraction of solvent molecules is about 26%. To investigate recrystallization, the solvent beads were subsequently removed, and the systems were quenched to $T = 2.33\ u/k_B$ for $1.141\ M\tau$. Inter-species entanglements in which all four chain ends resided within crystalline domains were classified as entangled intercrystallite loops.

Simulated tensile tests

To qualitatively assess the effect of entangled intercrystallite loops on mechanical behavior, we simulated tensile tests on the neat and annealed samples at a constant temperature $T = 2.33\ u/k_B$. The tensile deformation was applied along $z-$ axis at a constant rate of $\dot{L_z} = 0.0044a/\tau$, with the dimensions along the $x-$ and $y-$ axes remaining unchanged. The deformation rate is about 0.88 thermal velocity ($\sqrt{3k_B\mathrm{T}/m_b}$,) in the polymer sample. Given that the thermal velocity in PE at room temperature is of order about 1000 m/s, our CG tensile deformation rate in real units should be about 3.87 m/s, much faster than the deformation rates used in our experiments. The fast deformation rate, however, is inevitable in molecular simulations due to their accessible spatiotemporal scales.

Because our CG model treats crystalline chains as straight rods, the interchain friction within the crystalline phase is relatively small. Together with the fast tensile deformation rate, the reduced molecular frictions in the CG samples can lead to easy chain pullout from the crystalline domains. In contrast, real semicrystalline polymers such as *i*PP and PE possess zigzag backbones and side chains, which hinder continuous chain pullout during deformation.[8] To prevent chain pullout from the crystalline domains, we identified the crystalline atoms of polymers A and B and set their mutual interaction strength to $\epsilon_{CC} = 30$ while keeping all other interactions unchanged. The enhanced intermolecular interactions between crystalline atoms are sufficient to prevent polymer sliding within the crystalline domains.

To suppress chain crossing during deformation, the bond stretching spring constant $k_0$was increased to $3175u_0/a^2$. The tensile stress was calculated from the pressure tensor component $-P_{zz}$, and the strain was defined as $\lambda = L_z/L_z^0 - 1$. Each stress–strain curve represents an average over six independent tensile simulations initiated from different sample configurations.

We employed our previously established bond-breaking criteria,[9] defined as 150 times the maximum Lennard–Jones attractive force between non-crystalline atoms ($f_{break} = 220u_0/a$). To exclude apparent bond stretching caused by the enhanced crystalline–crystalline interactions, we calculated the average bond length for bonds connected to each atom. Atoms whose average bond length exceeds the defined breaking threshold are identified as “breaking points”, such as the pink highlights in **Figure 3B**. Bond-breaking events were tracked in each simulation frame and chains involved in such events were identified. If a given chain exhibits bond breaking in three consecutive frames, that chain and the kinks to which it is connect are classified as broken. For each tensile simulation, we recorded the maximum strain of samples at which all kinks bridging the interface become either fully broken or disentangled. The averaged values are reported in **Table S6**.

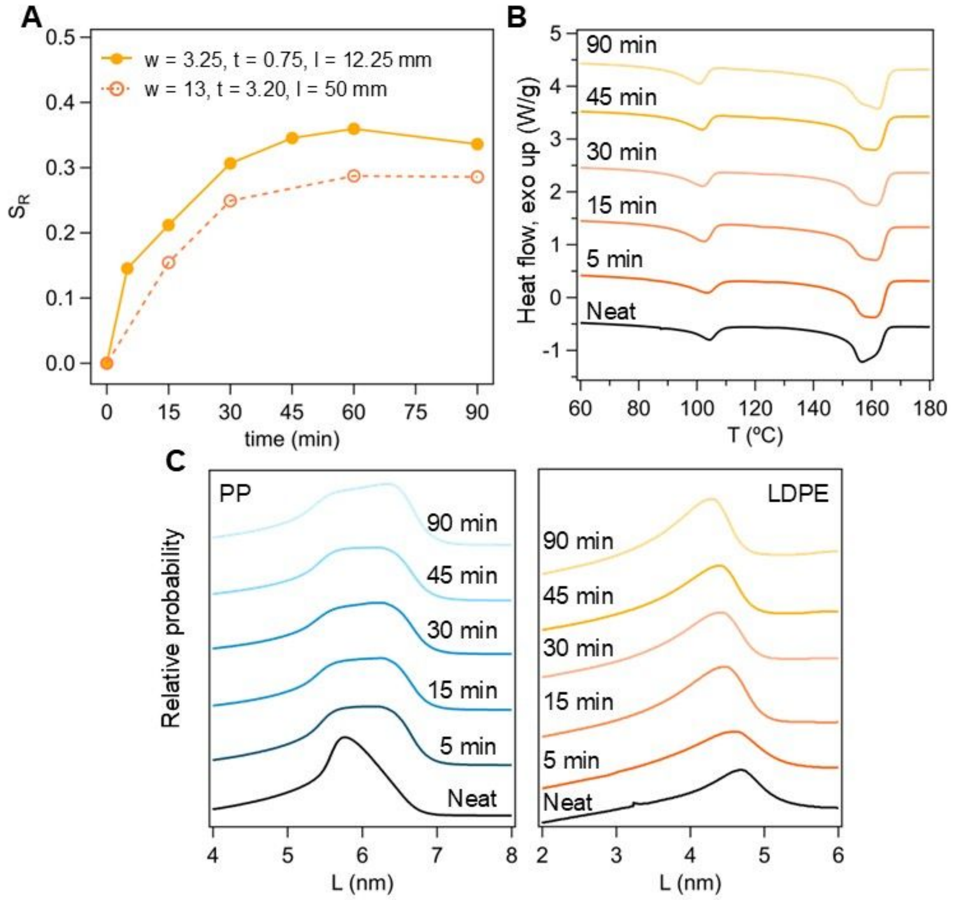

Figure S1. (**A**) Dimensional swelling ratio of 70PP/30LDPE at 80 °C as a function of swelling time for films (w = 3.25 mm, t = 0.75 mm, l = 12.25 mm) and injection molded bars (w = 13 mm, t = 3.2 mm, l = 50 mm) (**B**) Differential Scanning Calorimetry (DSC) thermograms of 70PP/30LDPE after thermal solvent immersion annealing at 80 °C at increasing times. (**C**) Lamellae thickness distributions of 70PP/30LDPE as a function of swelling time at 80 °C for both PP and PE domains.

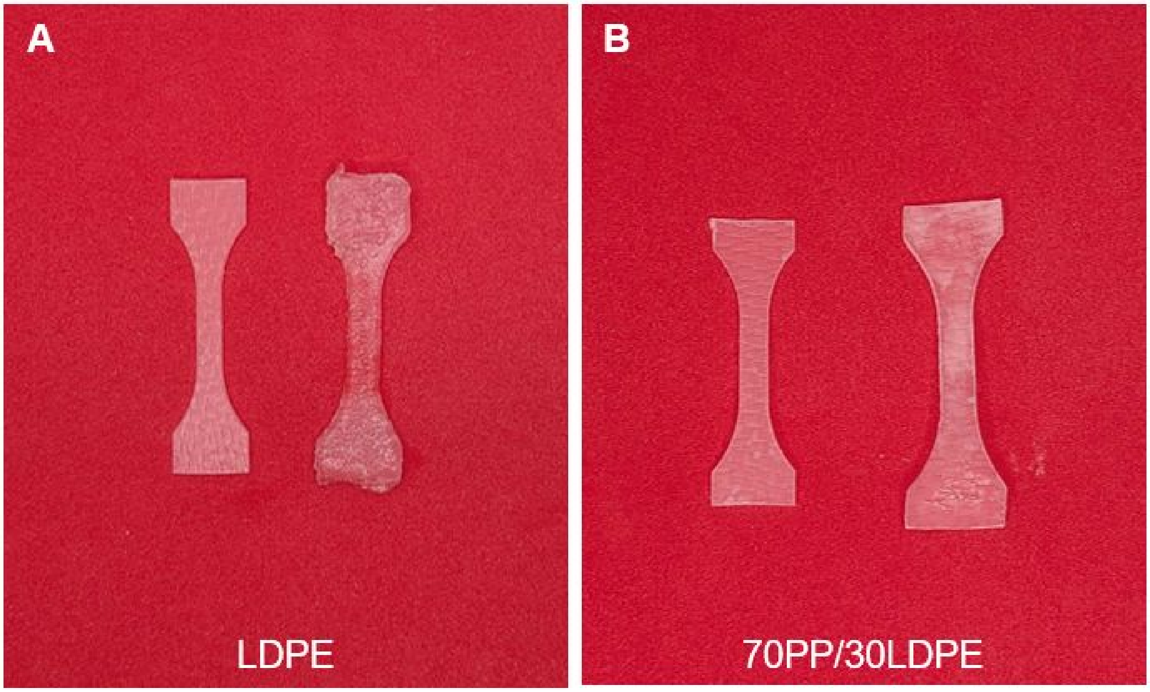

Figure S2. Images of (**A**) LDPE and (**B**) 70PP/30LDPE before (left) and after (right, swollen state) annealing at 80 °C xylenes for 1 h, demonstrating dimensional instability at elevated xylenes temperatures.

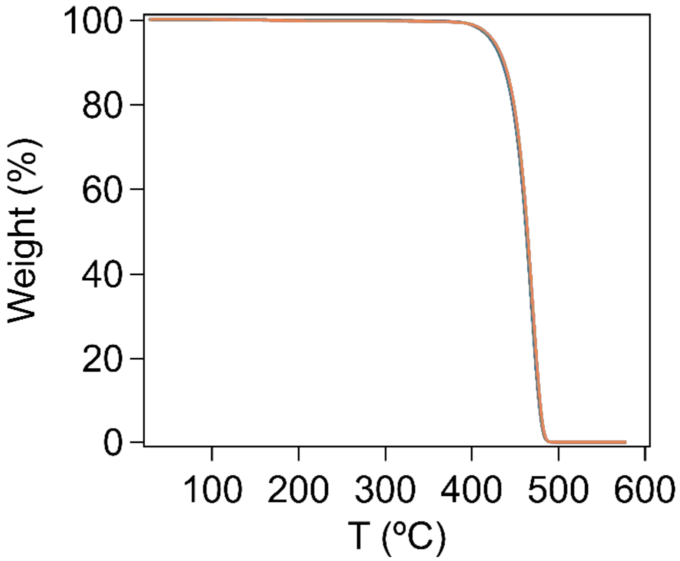

Figure S3. Thermogravimetric analysis of 70PP/30LDPE before (blue) and after (orange) thermal solvent immersion annealing and subsequent drying.

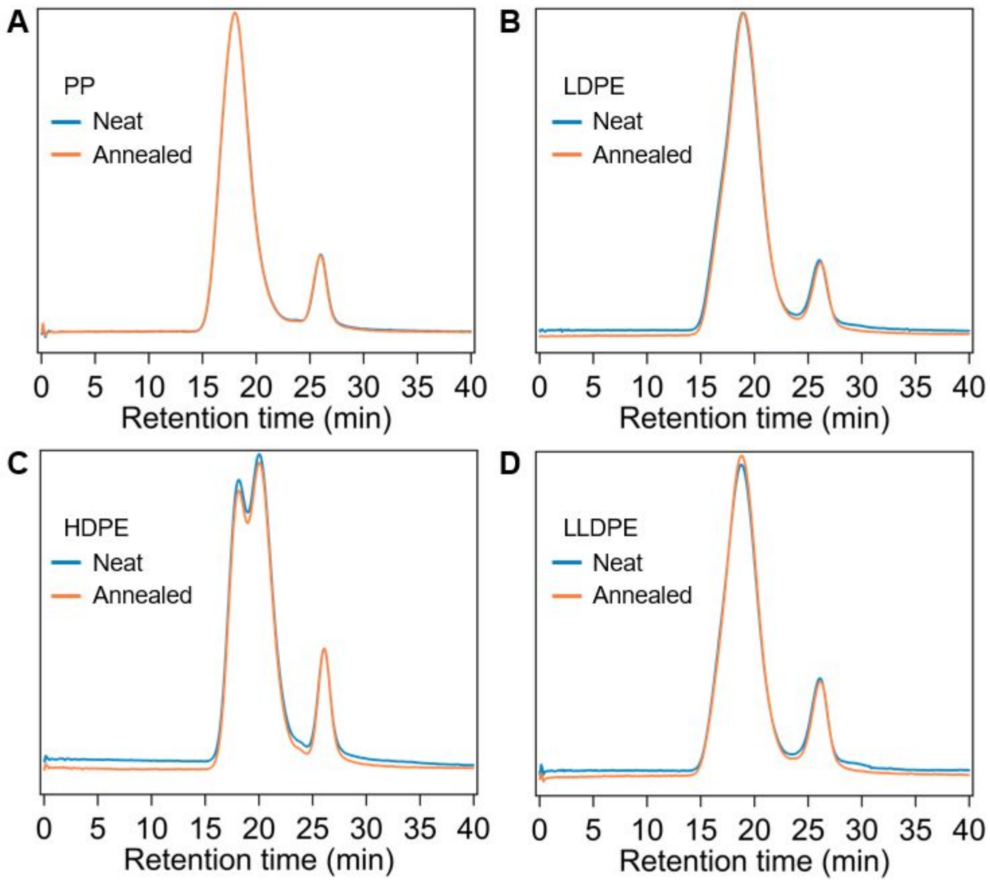

Figure S4. High temperature gel permeation chromatography of (**A**) PP, (**B**) LDPE, (**C**) HDPE, (**D**) LLDPE before (blue) and after (orange) thermal solvent immersion annealing.

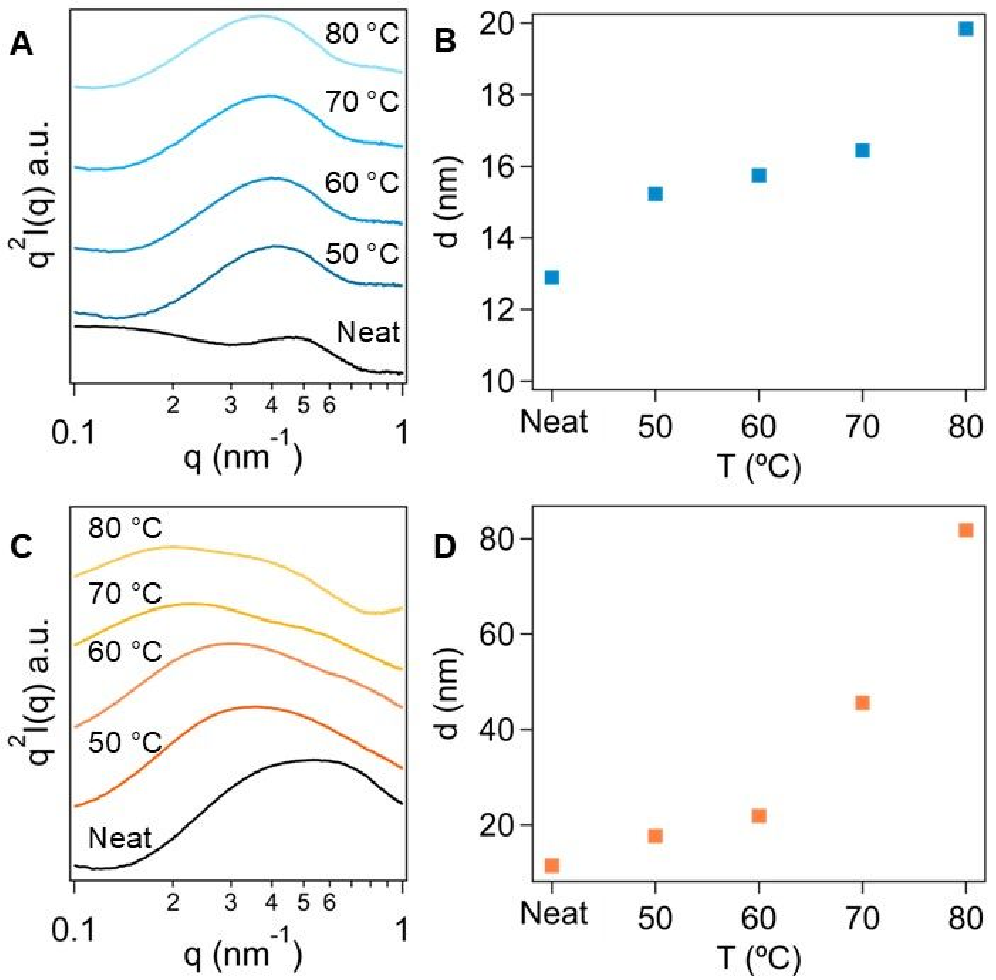

Figure S5. (**A**) PP *in situ* SAXS 1D profiles and (**B**) calculated long period (*d*) as a function of annealing temperature. (**C**) LDPE *in situ* SAXS 1D profiles and (**D**) calculated long period (*d*) as a function of annealing temperature.

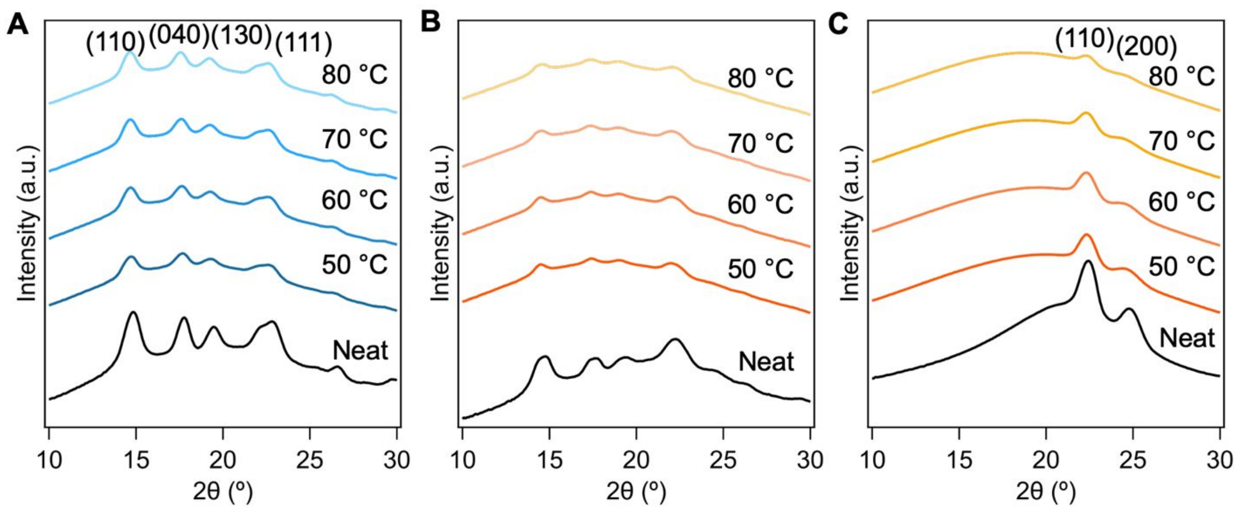

Figure S6. *In situ* 1D WAXS profiles of (**A**) PP, (**B**) 70PP/30LDPE, and (**C**) LDPE in neat state and as a function of temperature during thermal solvent immersion annealing in xylenes. Characteristic Miller indices of PP and LDPE are labeled.

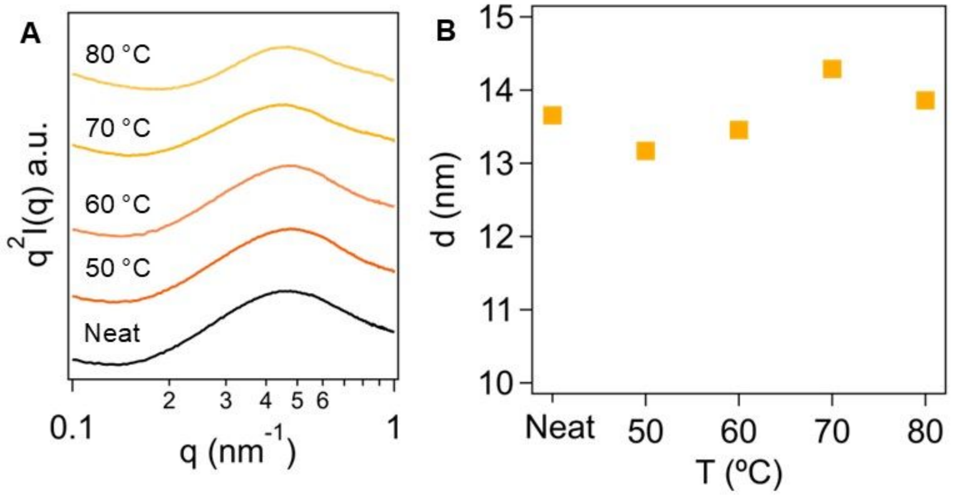

Figure S7. (**A**) 1D SAXS profiles of 70PP/30LDPE in neat and dried state after thermal solvent immersion annealing at different temperatures and (**B**) long period (*d*) as a function of annealing condition.

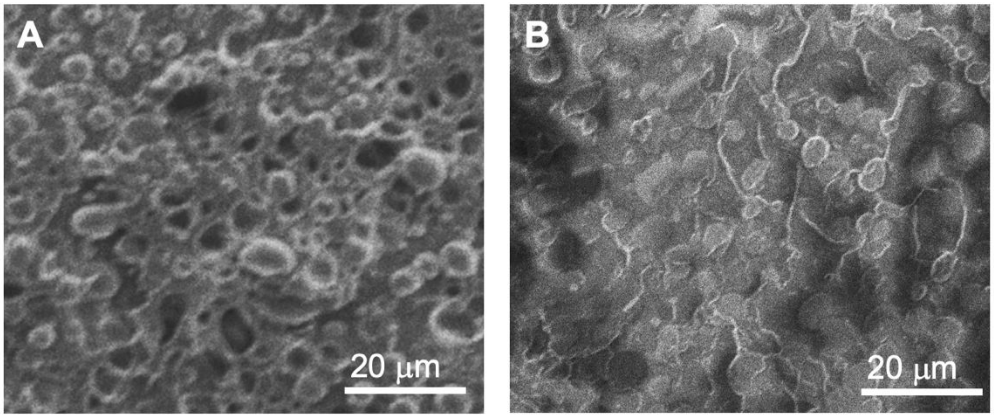

Figure S8. Scanning Electron Microscopy (SEM) images of 70PP/30LDPE (**A**) before and (**B**) after thermal solvent immersion annealing at 80 ˚C for 1 hour in xylenes.

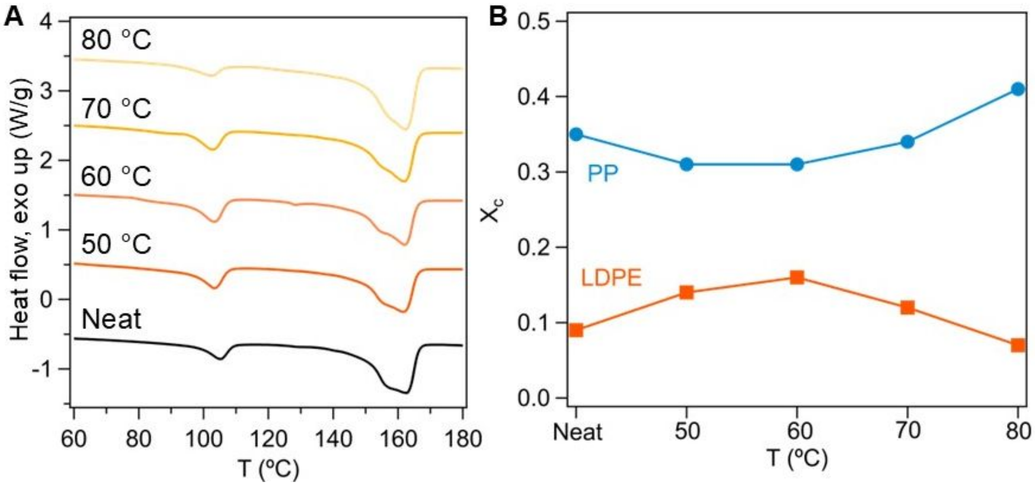

Figure S9. (**A**) DSC heating thermograms of 70PP/30LDPE before (neat) and after annealing at increasing temperatures and (**B**) Relative degree of crystallinity (X_c) of 70PP/30LDPE blends before (neat) and after annealing at different temperatures for 1 hour.

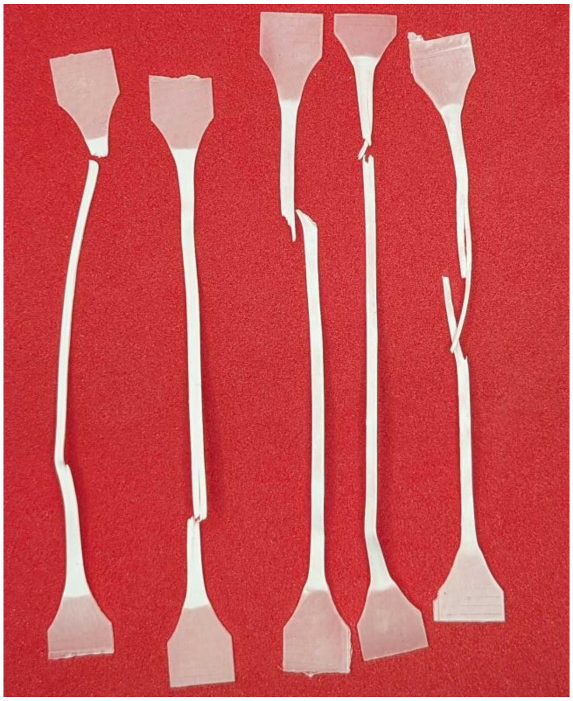

Figure S10. Image of tensile replicates of 70PP/30LDPE after thermal solvent immersion annealing in xylenes at 80 ˚C for 1 hour.

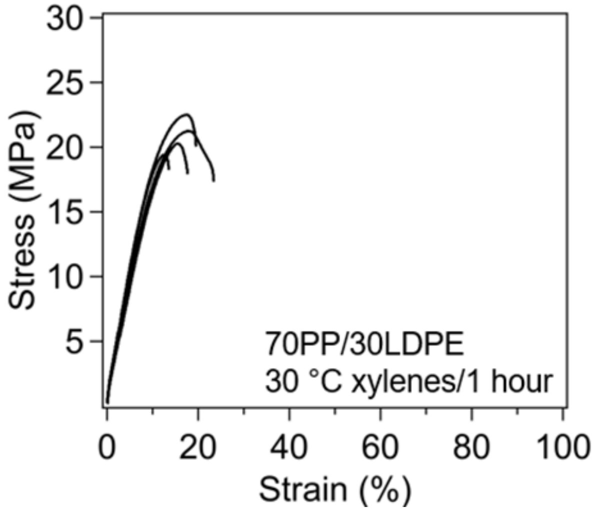

Figure S11. Stress-strain curves of 70PP/30LDPE after thermal solvent immersion annealing at 30 ˚C for 1 hour without drying.

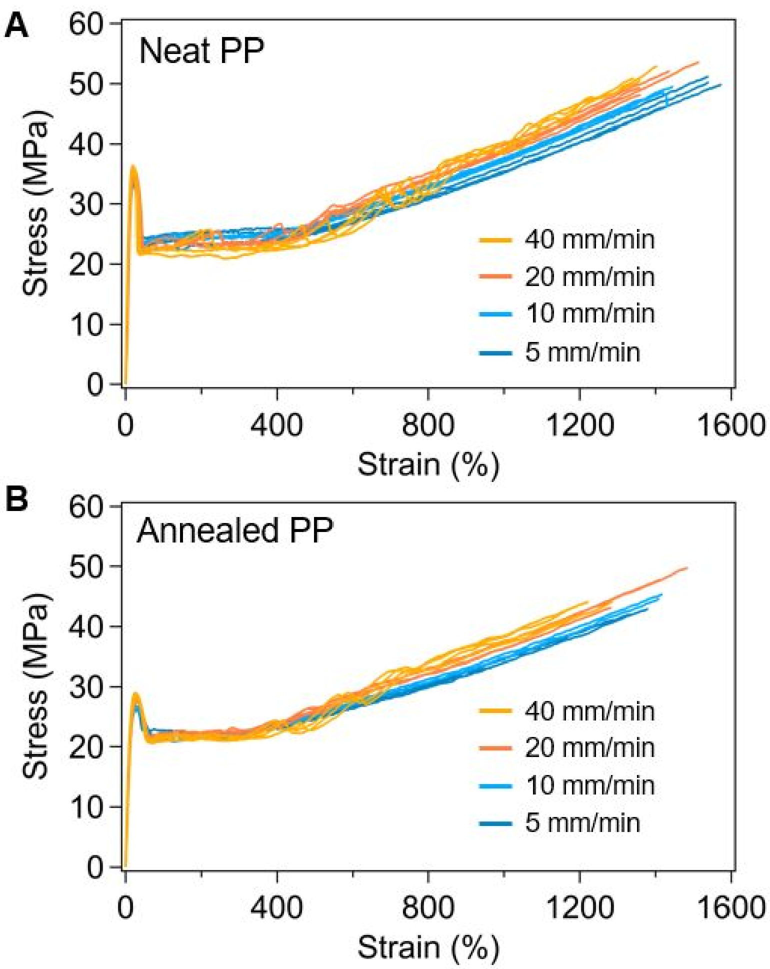

Figure S12. Stress-strain curves of (**A**) Neat PP and (**B**) PP after thermal solvent immersion annealing at 80 °C for 1 hour tested at different strain rates.

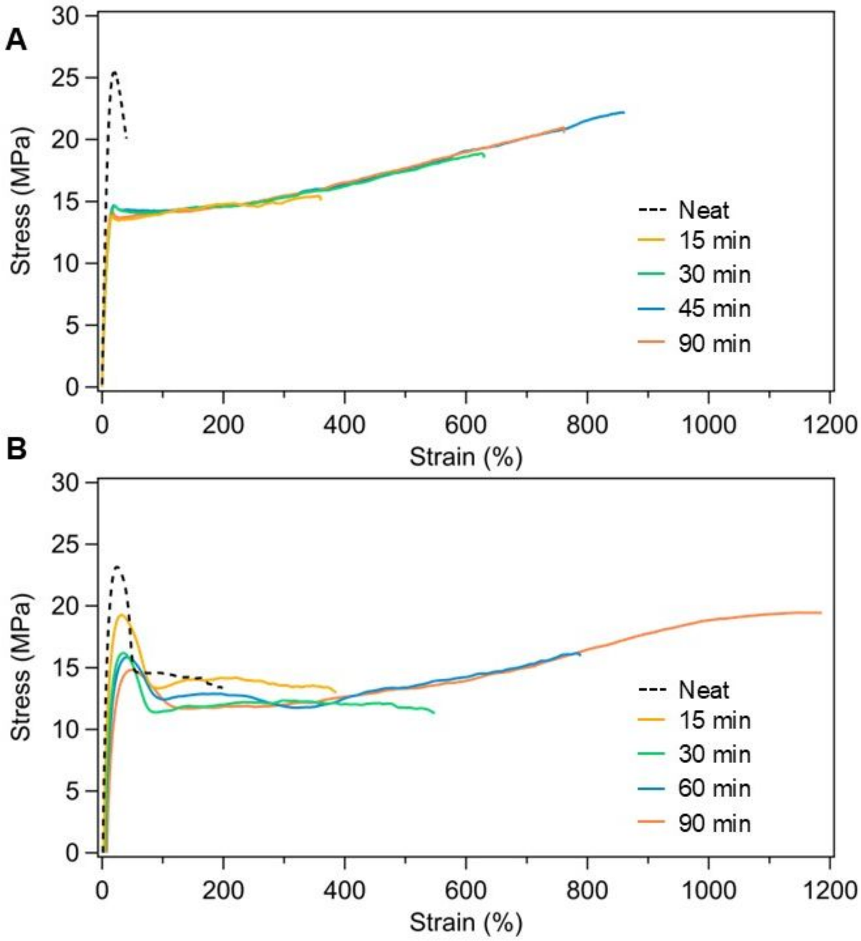

Figure S13. Stress-strain curves of 70PP/30LDPE with increasing thermal immersion annealing time at 80 ˚C for (**A**) films (w = 3.25, t = 0.75, l = 12.25 mm) and (**B**) injection molded bars (w = 13, t = 3.20, l = 50 mm).

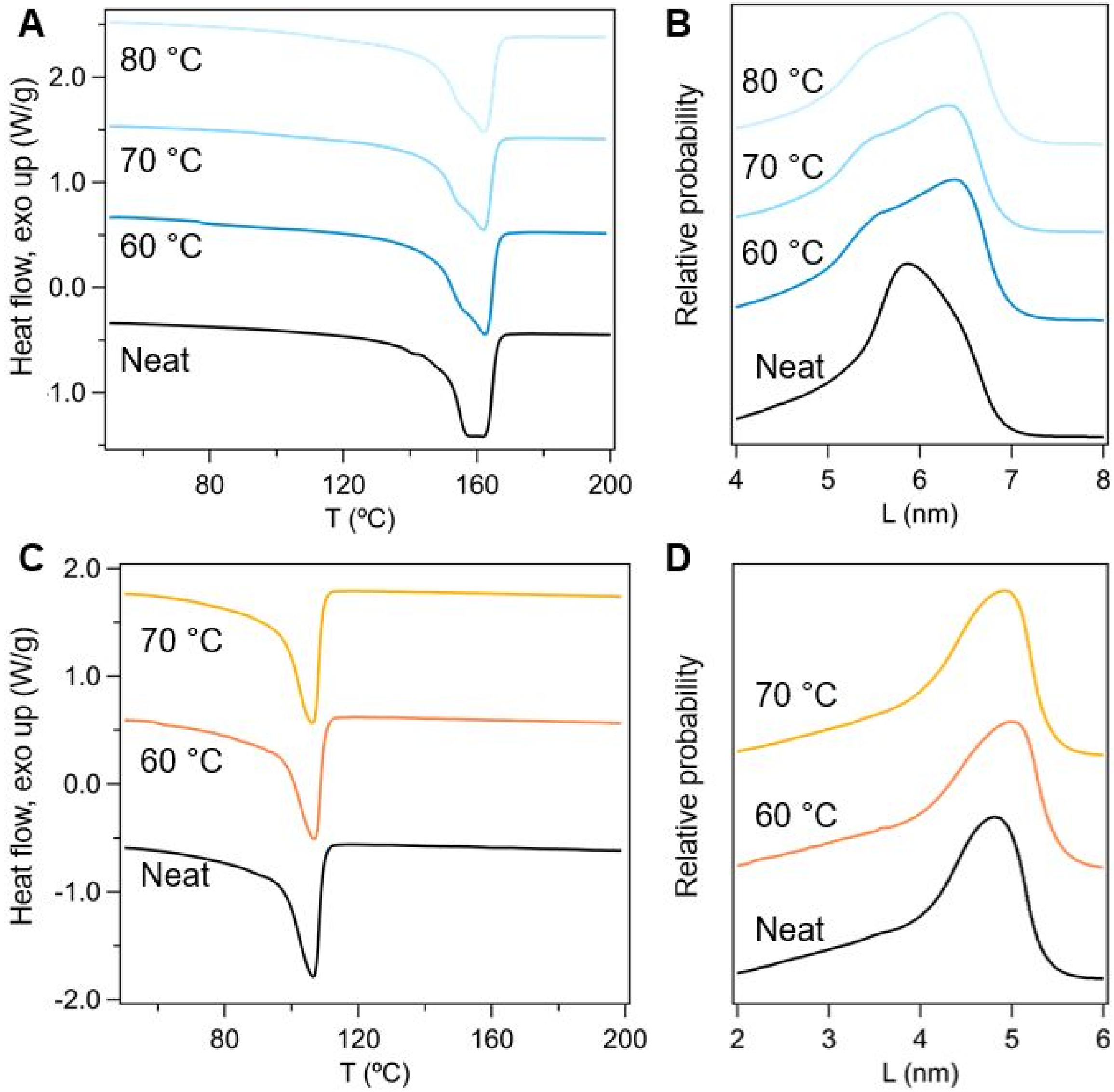

Figure S14. (**A**) DSC thermograms and (**B**) lamellae thickness distributions of PP homopolymer before (Neat) and after thermal solvent immersion annealing at increasing temperatures. (**C**) DSC thermograms and (**D**) lamellae thickness distributions of LDPE homopolymer before (neat) and after thermal solvent immersion annealing at different temperatures.

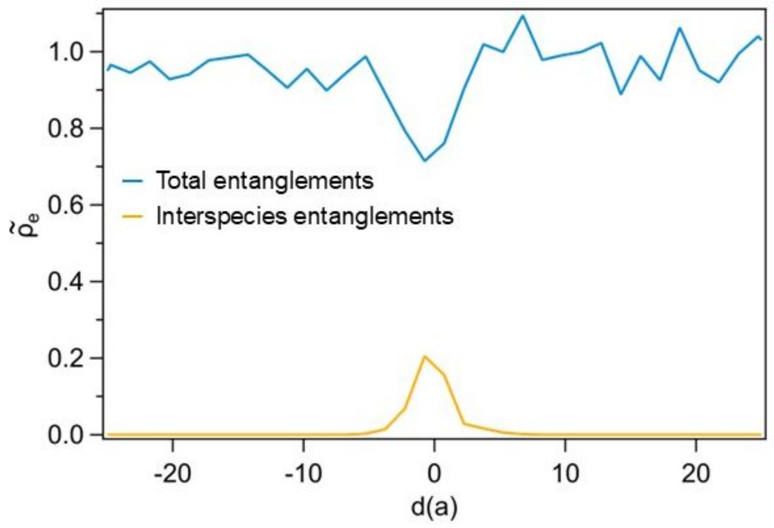

Figure S15. Normalized total and interspecies entanglement densities near a non-compatibilized semicrystalline interface.

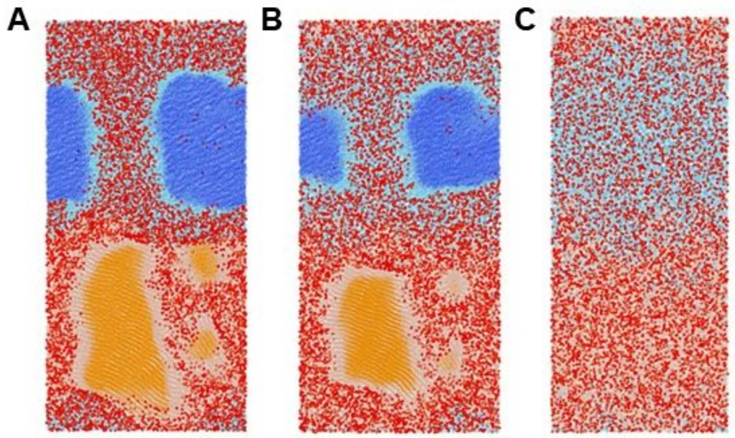

Figure S16. Snapshots of (**A**) Anneal-L, (**B**) Anneal-M, and (**C**) Anneal-F during simulated thermal solvent immersion annealing. The solvent molecules are colored in red. As the restraints applied to crystalline atoms are relaxed, the solvent molecules penetrate and the crystals can be melt.

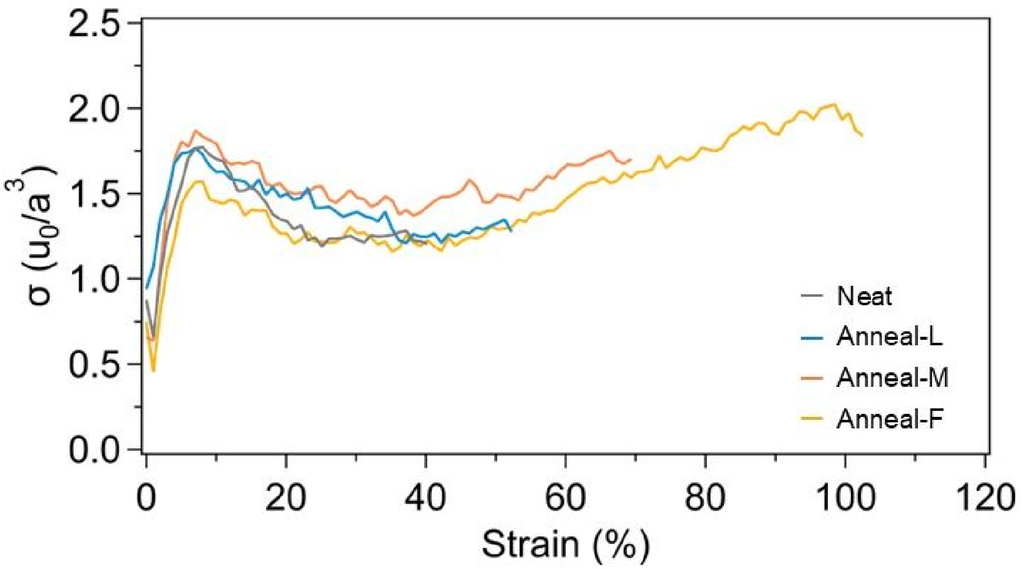

Figure S17. Simulated stress–strain curves for semicrystalline interfaces quenched from the melt, where different fractions of crystalline atoms were restrained.

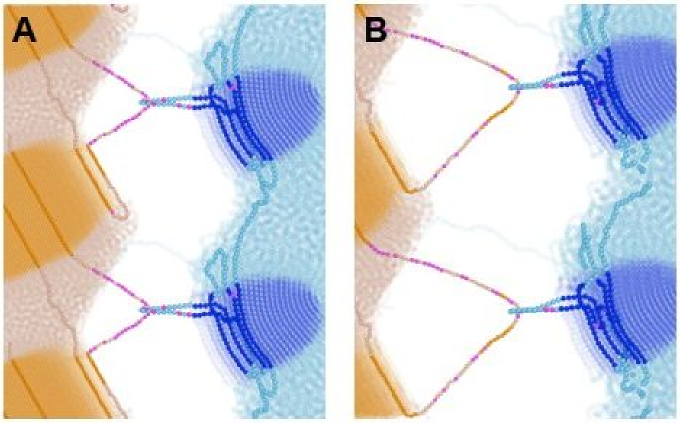

Figure S18. Snapshots of neat samples during simulated tensile tests at (**A**) 30% strain and (**B**) 40% strain beyond the point of failure. Bonds possessing forces which exceed the bond break threshold are shown in pink.

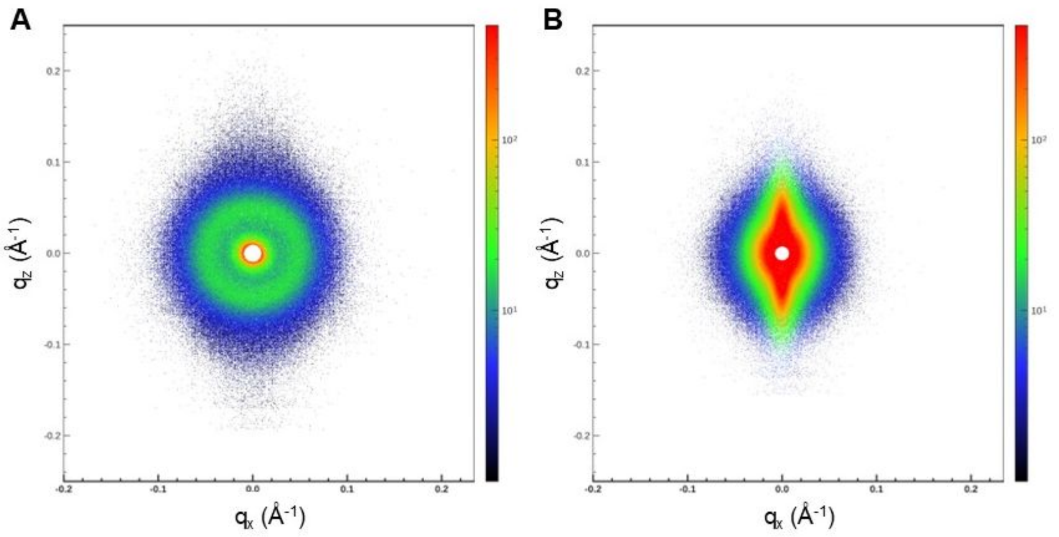

Figure S19. 2D SAXS patterns of thermal solvent immersion annealed 70PP/30LDPE at (**A**) 0 % strain and (**B**) 300 % strain. The central beam region was masked, and the data in the q range of 0.0075-0.2 Å^{-1} are shown.

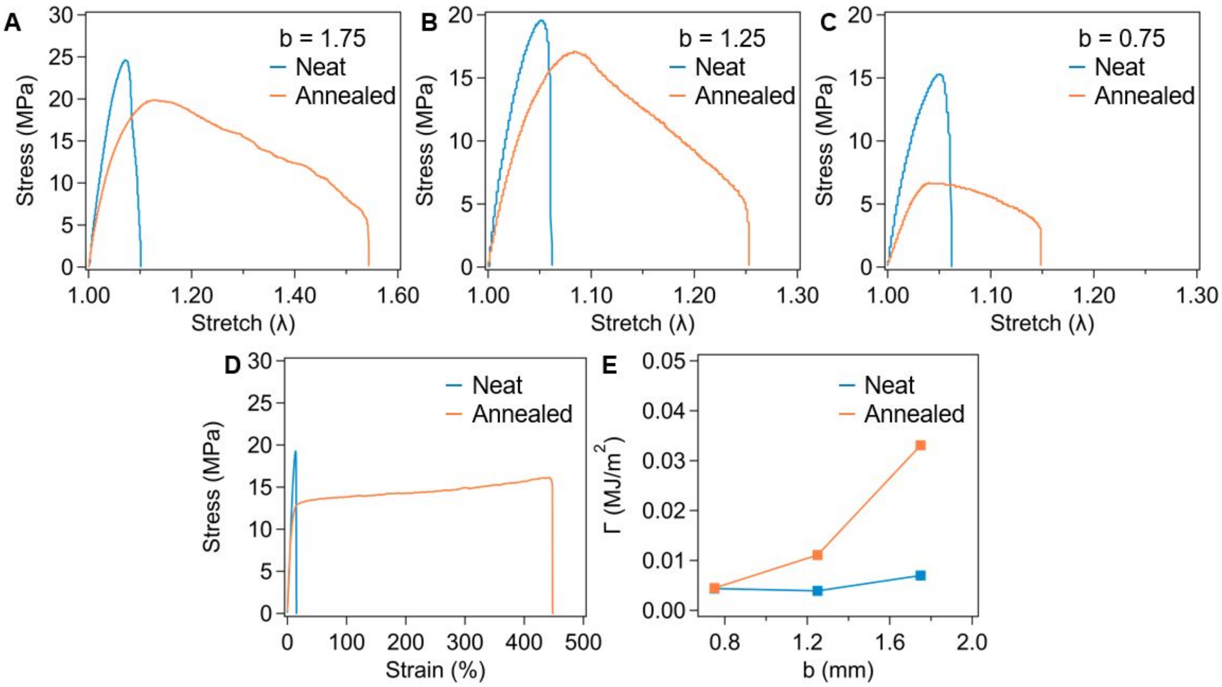

Figure S20. Stress-stretch curves of 70PP/30LDPE before (blue) and after (orange) thermal solvent immersion annealing with ligament length (b) of (**A**) 1.75 mm (**B**) 1.25 mm (**C**) 0.75 mm; (**D**) stress-strain curves of 70PP/30LDPE before (blue) and after (orange) thermal solvent immersion annealing (crosshead displacement of 5 mm/min, unnotched bars) and (**E**) fracture toughness (Γ)

as a function of b for 70PP/30LDPE blends before (blue) and after (orange) thermal solvent immersion annealing.

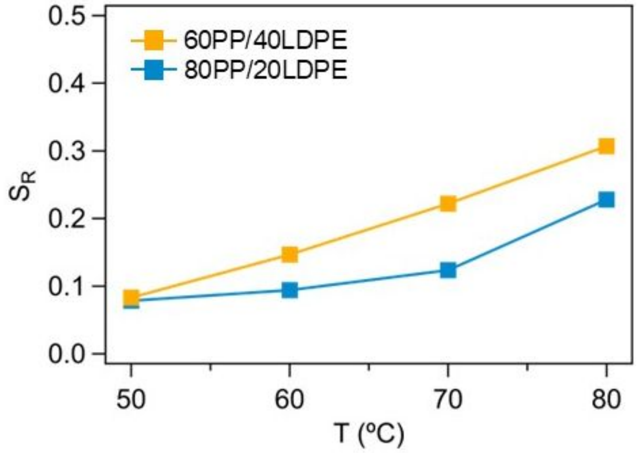

Figure S21. Swelling ratios (S_R) of 80PP/20LDPE and 60PP/40LDPE at different annealing temperatures.

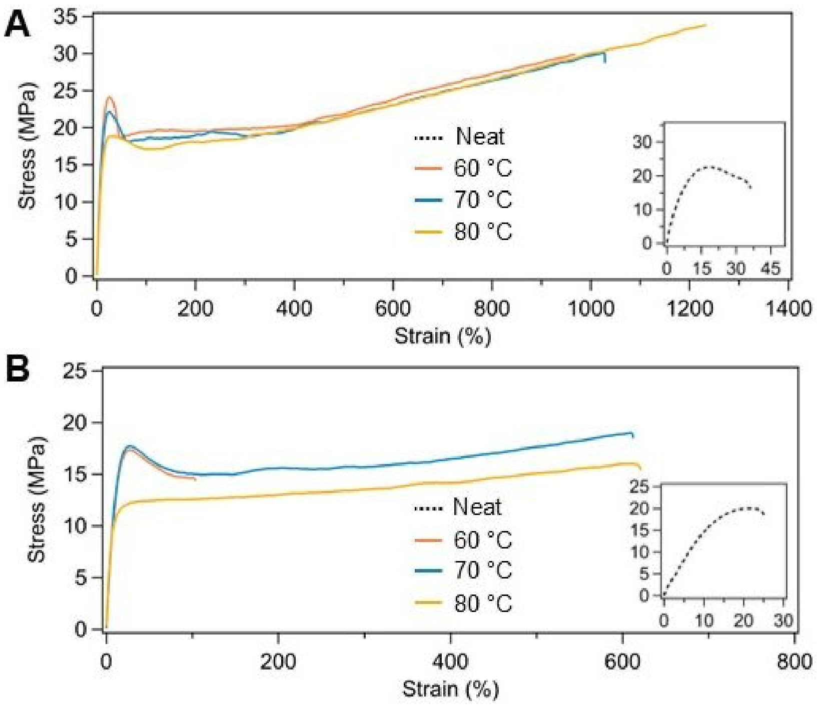

Figure S22. Stress-strain curves of (**A**) 80PP/20LDPE and (**B**) 60PP/40LDPE before (dashed line) and after thermal solvent immersion annealing (solid lines) at increasing temperature in xylenes for 1 hour.

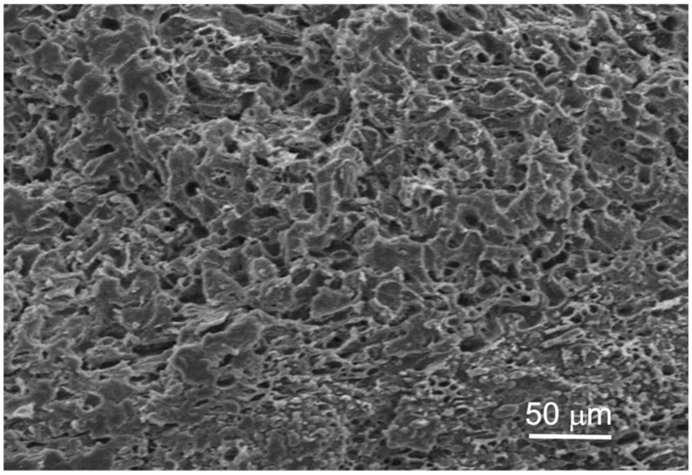

Figure S23. SEM image of 60PP/40LDPE after thermal solvent immersion annealing at 80 ˚C for 1 hour in xylenes.

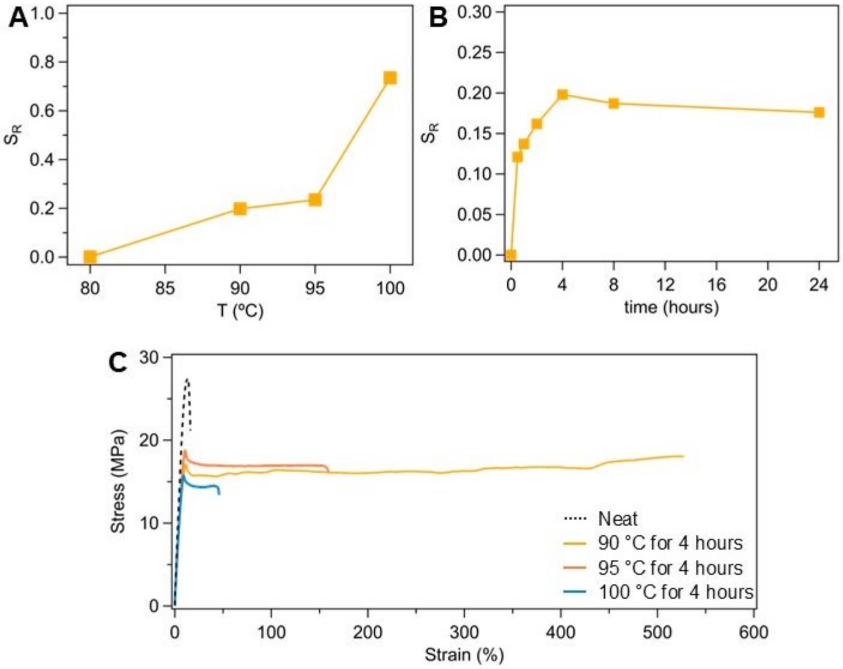

Figure S24. (**A**) Swelling ratio of 70PP/30HDPE as a function of temperature, (**B**) swelling kinetics of 70PP/30HDPE as a function of time in xylenes at 90 ˚C, and (**C**) stress-strain curves of 70PP/30HDPE as a function of annealing temperature after equilibrium swelling is achieved (4 hours).

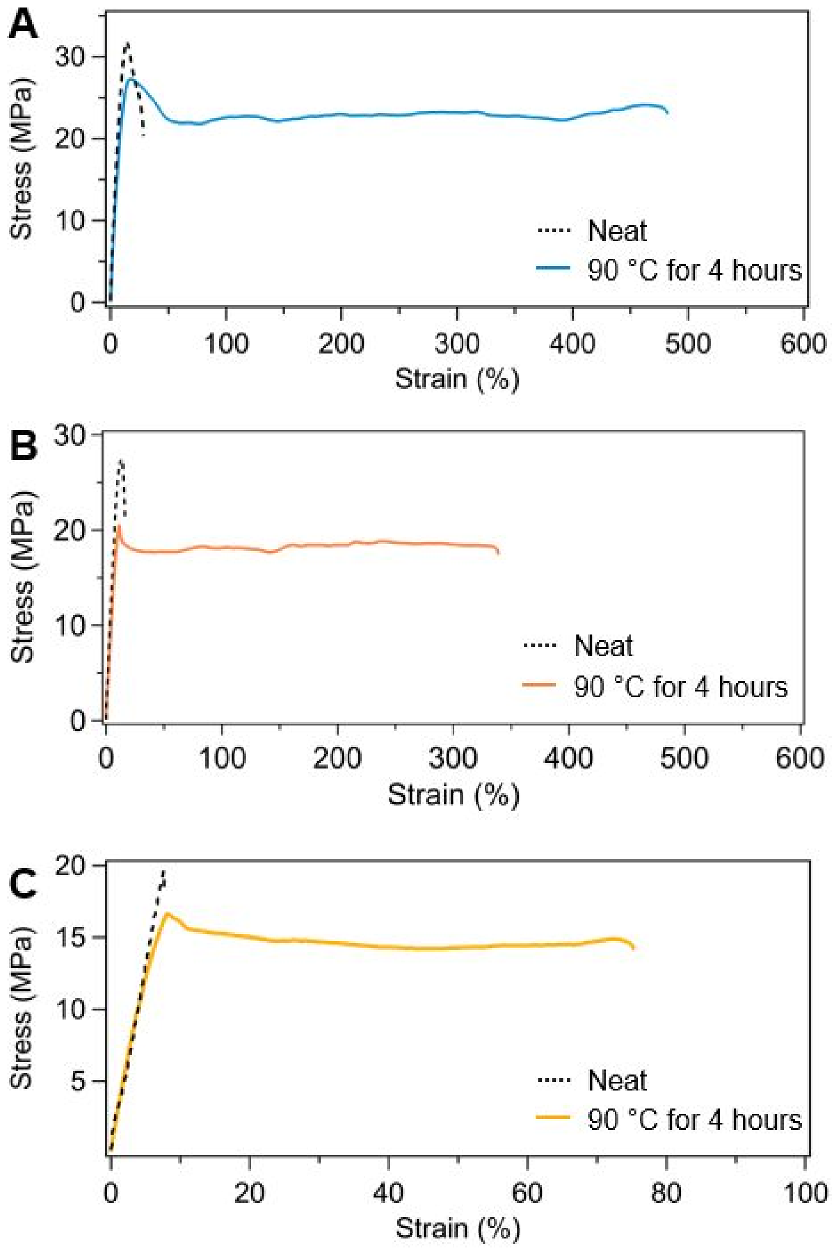

Figure S25. Stress-strain curves of (**A**) 85PP/15HDPE, (**B**) 70PP/30HDPE, and (**C**) 60PP/40HDPE before and after thermal solvent immersion annealing in xylenes at 90 ˚C for 4 hours.

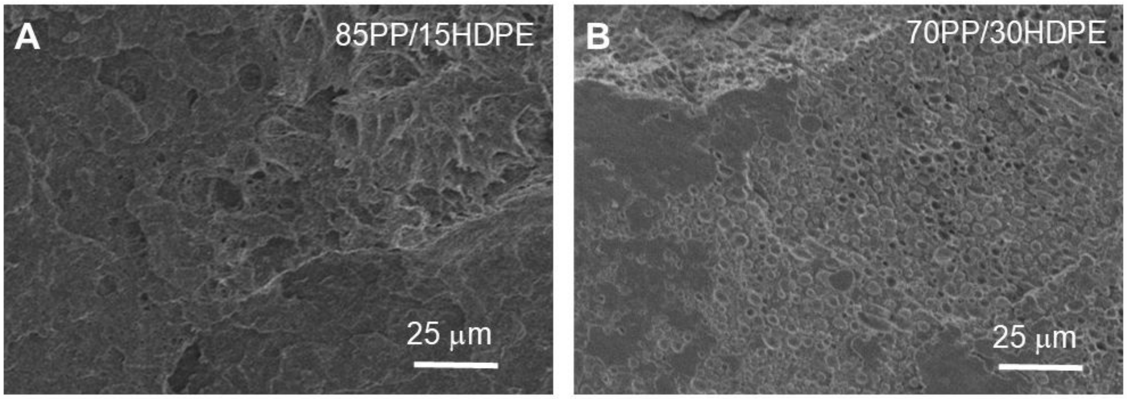

Figure S26. SEM images of (**A**) 85PP/15HDPE and (**B**) 70PP/30HDPE after thermal solvent immersion annealing at 90 ˚C for 4 hours in xylenes.

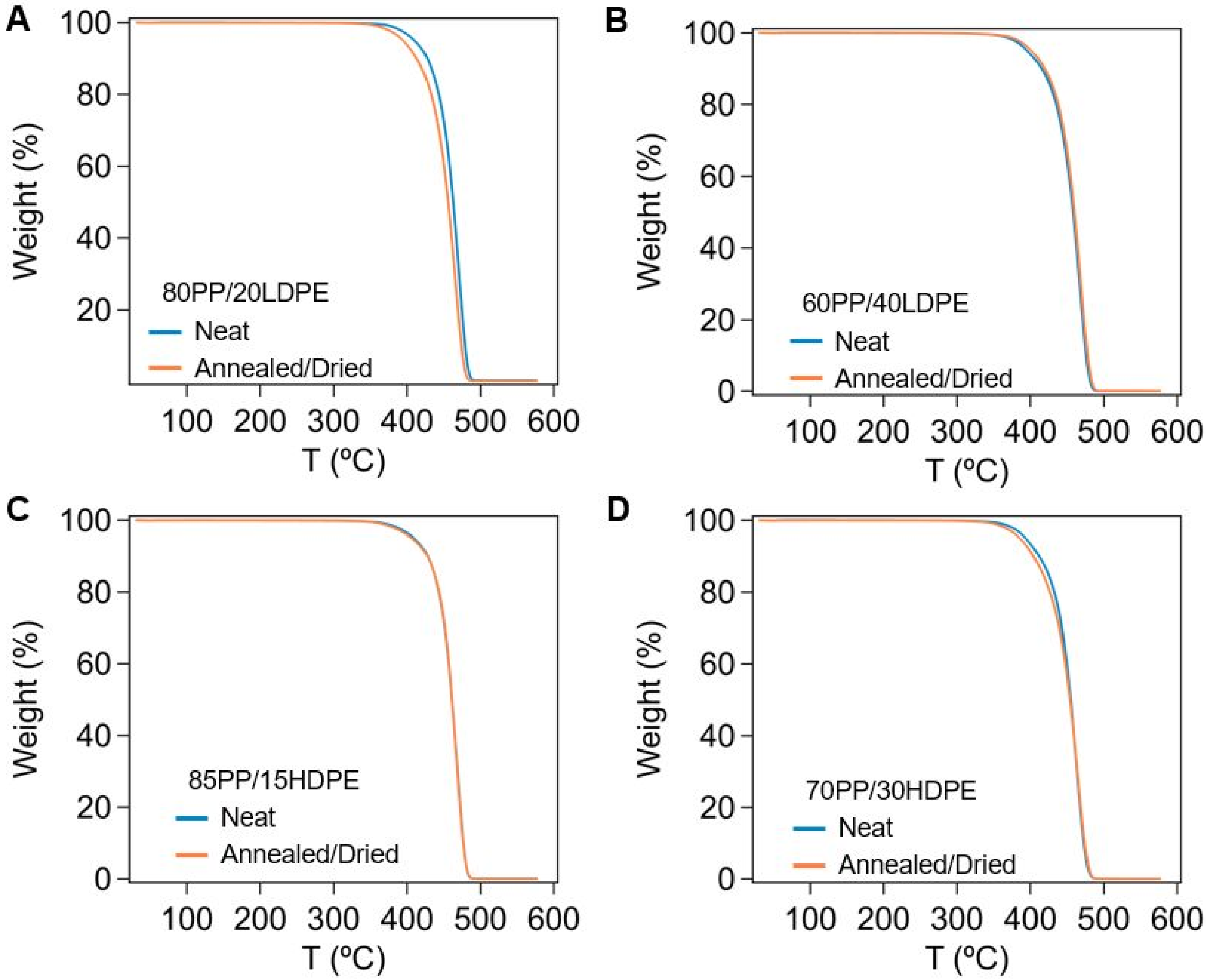

Figure 27. TGA of (**A**) 80PP/20LDPE, (**B**) 60PP/40LDPE, (**C**) 85PP/15HDPE, and (**D**) 70PP/30HDPE before (blue) and after (orange) thermal solvent immersion annealing and subsequent drying.

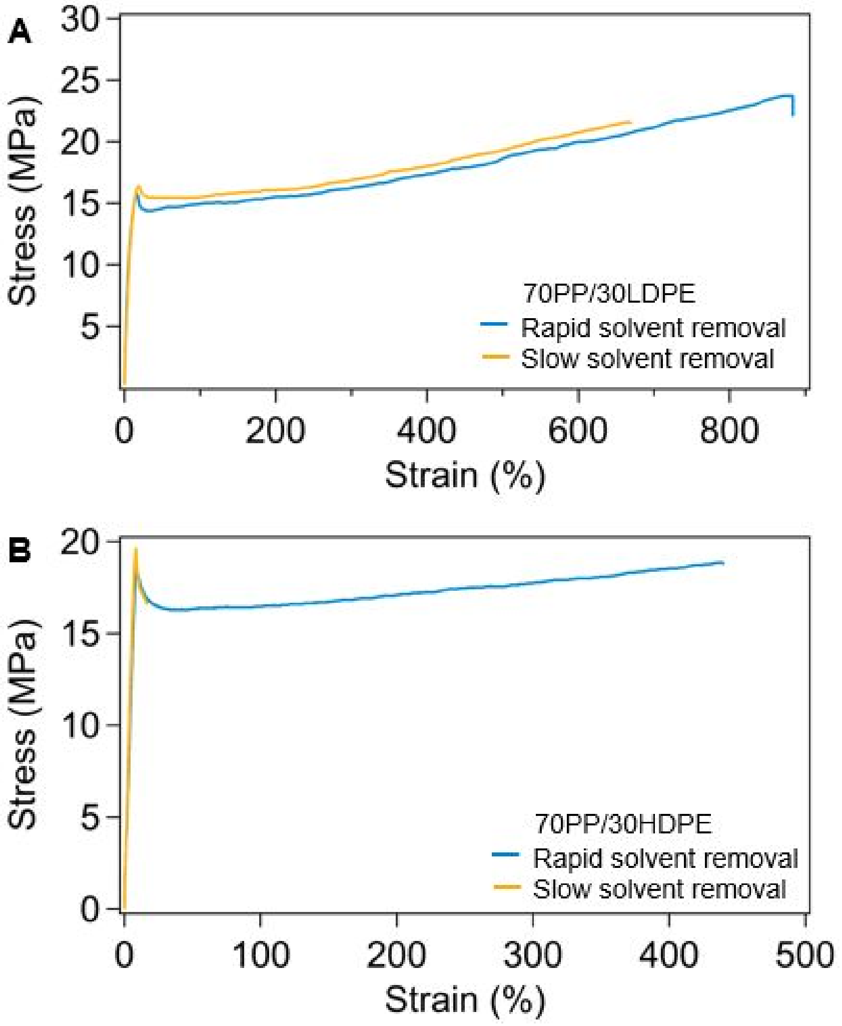

Figure S28. Stress-strain curves of (**A**) 70PP/30LDPE and (**B**) 70PP/30HDPE after thermal solvent immersion annealing and rapid solvent removal in blue (specimens pulled from solvent bath directly to cool) and slow solvent removal in yellow (specimens cooled in the solvent bath).

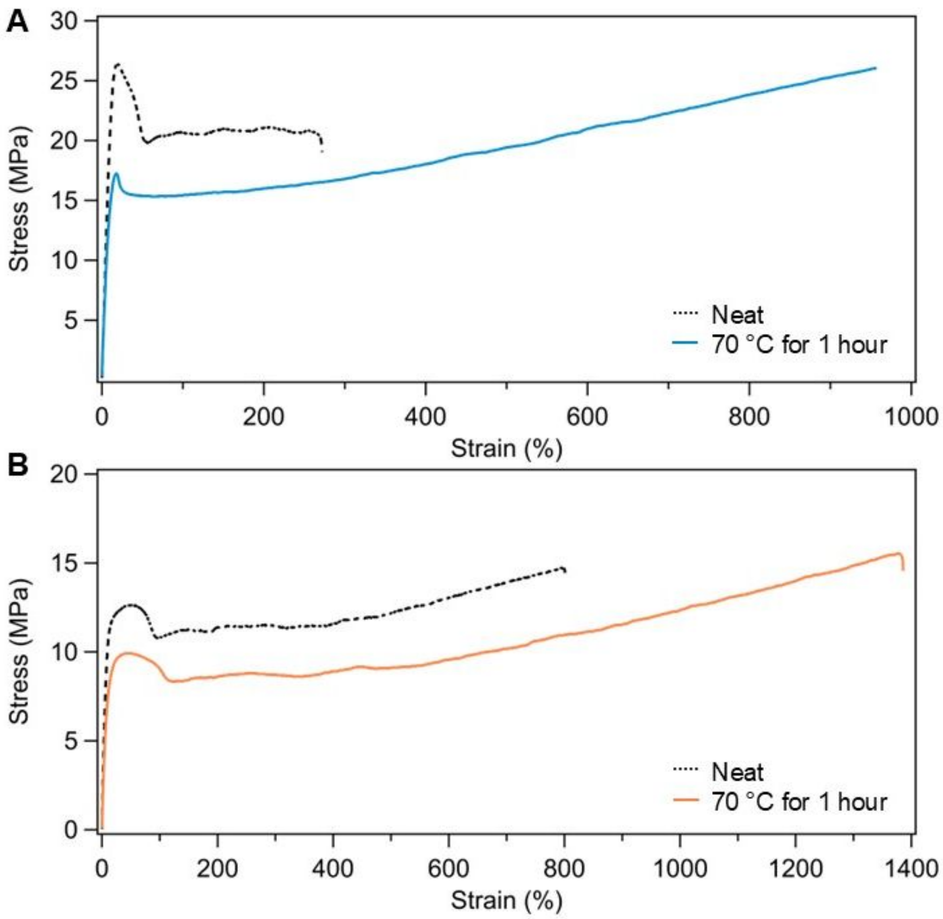

Figure S29. Stress-strain curves of (**A**) 70PP/30LLDPE and (**B**) 30PP/70LLDPE before (dashed) and after (solid) thermal solvent immersion annealing.

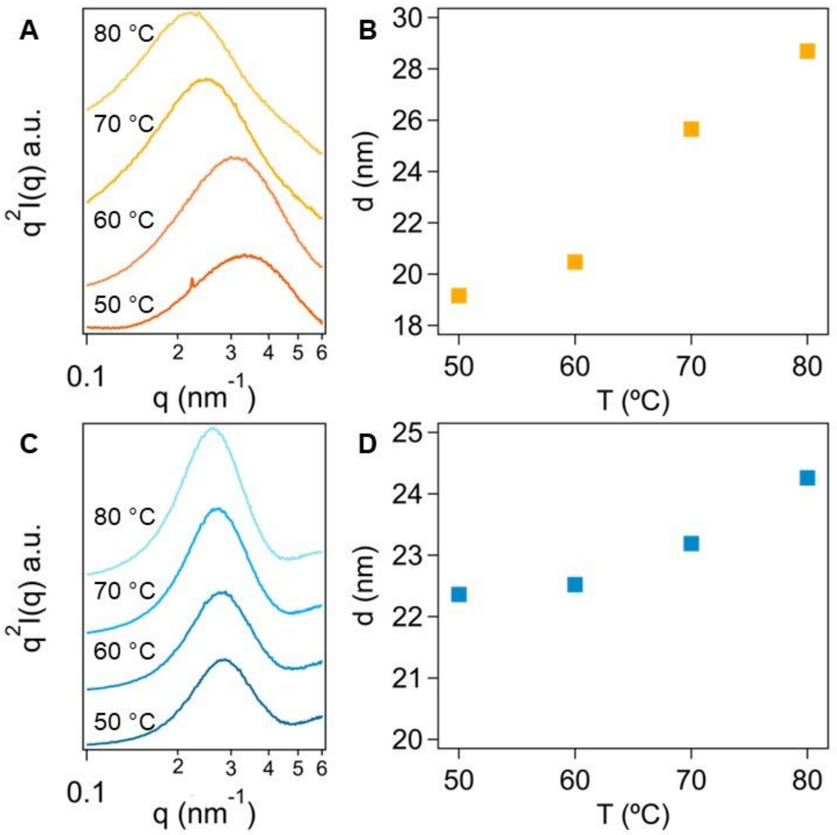

Figure S30. (**A**) *in situ* 1D SAXS profiles and (**B**) long period (*d*) of LLDPE as a function of annealing temperature in xylenes. (**C**) *In situ* 1D SAXS profiles and (**D**) *d* of HDPE as a function of annealing temperature in xylenes.

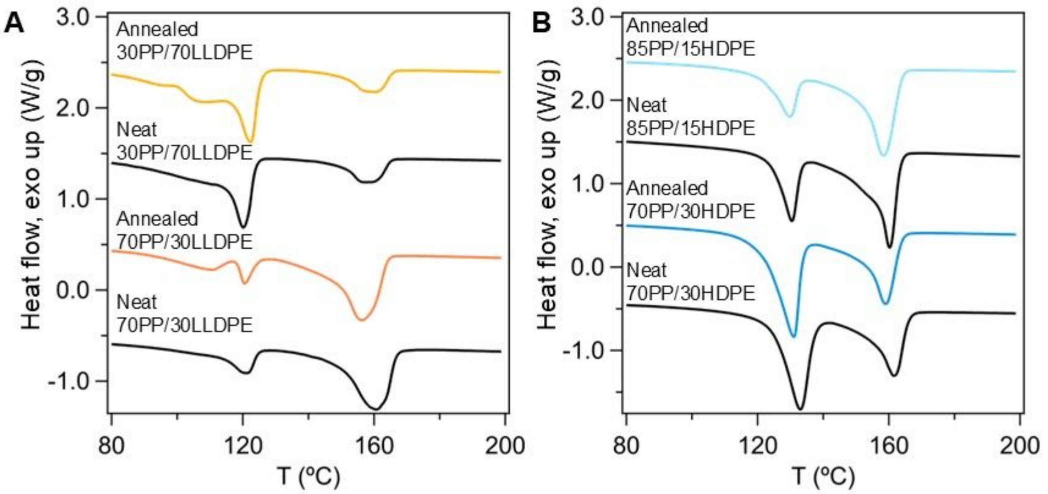

Figure S31. DSC thermograms of (**A**) 70PP/30LLDPE and 30PP/70LLDPE and (**B**) 85PP/15HDPE and 70PP/30HDPE before and after thermal immersion annealing.

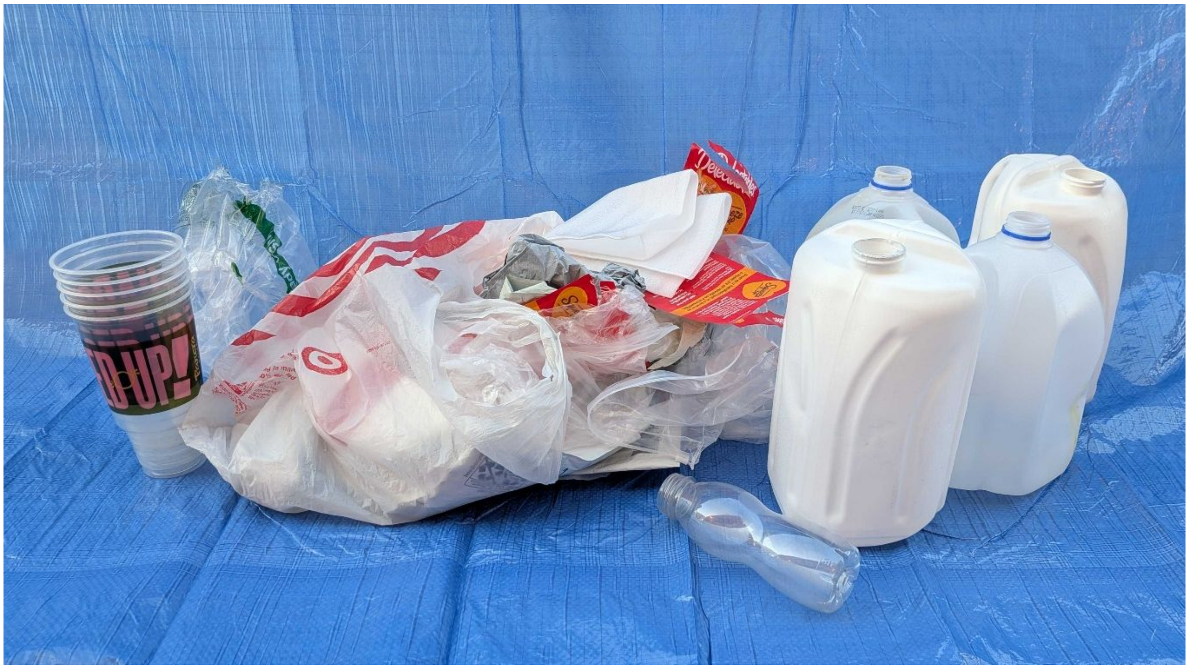

Figure S32. Image of post-consumer polyolefin wastes collected and used for demonstrating the closed-loop mechanical recycling enabled by thermal solvent immersion annealing.

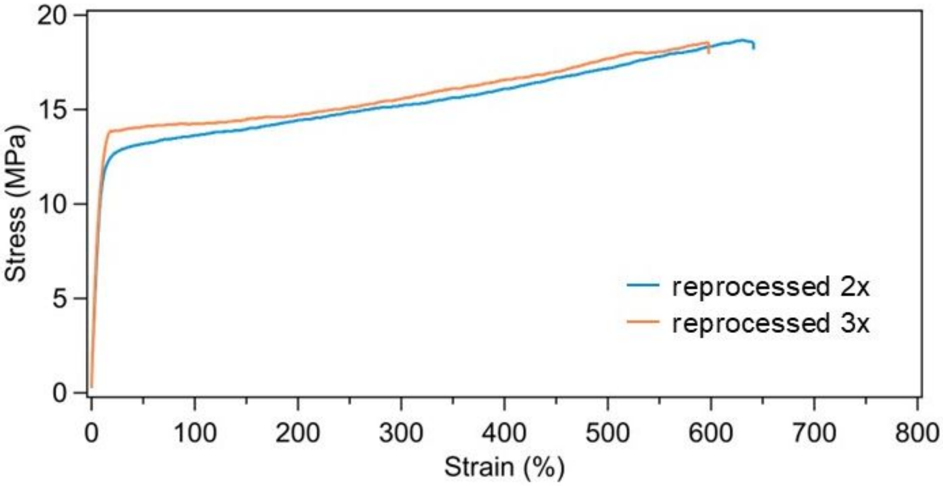

Figure S33. Stress-strain curves of 70PP/30LDPE after repeated melt reprocessing and thermal solvent immersion annealing cycles (at 80 ^{0}C in xylenes for 1 hour).

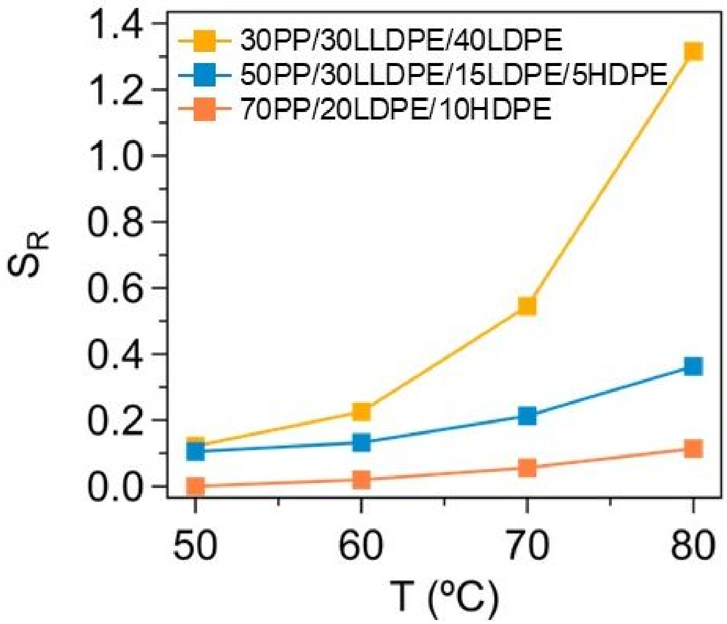

Figure S34. Swelling ratio (S_R) of ternary and quaternary PP/PE blends after annealing in xylenes at different temperatures.

Table S1. Mechanical properties of PP, LDPE, and 70PP/30LDPE before and after annealing at different temperatures.

Composition	Immersion Annealing T (ºC)	Elastic Modulus (MPa)	Toughness (MJ/m^3)	Strain at Break (%)	Stress at Break (MPa)
PP	Neat	430 ± 5	427 ± 41	1326 ± 15	45.2 ± 5.6
	60	203 ± 13	419 ± 23	1415 ± 22	45.7 ± 1.4
	70	271 ± 19	428 ± 27	1365 ± 9	49.1 ± 1.9
	80	203 ± 8	425 ± 46	1378 ± 18	46.9 ± 3.2
70PP/30LDPE	Neat	254 ± 13	16 ± 3	88 ± 14	18.2 ± 2.0
	60	211 ± 9	70 ± 3	408 ± 17	16.5 ± 0.8
	70	214 ± 10	105 ± 11	596 ± 56	17.8 ± 1.5
	80	242 ± 18	144 ± 11	807 ± 46	21.9 ± 0.3
LDPE	Neat	80 ± 1	94 ± 2	904 ± 1	13.9 ± 0.3
	60	54 ± 1	55 ± 5	683 ± 51	9.3 ± 0.2
	70	64 ± 6	45 ± 1	628 ± 4	7.7 ± 0.1

Table S2. Degree of crystallinity (X_c) and peak melting temperature taken from the first heat (T_m) of neat PP, LDPE, HDPE, and LLDPE.

Material	X_c*	T_m (˚C)*
PP	0.33	163.2
LDPE	0.21	107.5
LLDPE	0.30	121.6
HDPE	0.71	134.1

*Measurements collected from first heats of DSC scans

Table S3. Swelling ratio for PP and 70PP/30LDPE blends and corresponding sample mass before (m_i), immediately following (m_s) thermal solvent immersion annealing, and after drying (m_f).

Composition	Immersion Annealing T (˚C)	S_R	m_i (g)	m_s (g)	m_f (g)
PP	30	0.005	0.148	0.156	0.148
	40	0.030	0.146	0.168	0.145
	50	0.045	0.165	0.197	0165
	60	0.057	0.161	0.209	0.161
	70	0.086	0.192	0.238	0.191
	80	0.111	0.178	0.236	0.178
70PP/30LDPE	30	0.006	0.187	0.194	0.188
	40	0.078	0.163	0.176	0.162
	50	0.083	0.169	0.193	0.170
	60	0.094	0.146	0.168	0.147
	70	0.171	0.158	0.201	0.159
	80	0.359	0.182	0.287	0.181

Table S4. Crystalline properties of PP, LDPE, and 70PP/30LDPE before and after thermal immersion annealing.

Composition	Immersion Annealing T (°C)*	$T_{m,PP}$ (^{0}C)*	$T_{m,PE}$ (^{0}C)*	$X_{c,PP}$*	$X_{c,PE}$*
PP	Neat	163.2	-	0.33	-
	60	162.5	-	0.37	-
	70	162.0	-	0.32	-
	80	162.1	-	0.40	-
70PP/30LDPE	Neat	157.5	104.5	0.29	0.10
	60	160.6	102.1	0.28	0.18
	70	155.4	101.8	0.33	0.18
	80	160.2	99.7	0.33	0.18
LDPE	Neat	-	107.5	-	0.21
	60	-	106.8	-	0.24
	70	-	106.3	-	0.24

*Measurements collected from first heats of DSC scans

Table S5. Mechanical properties of PP tested at different strain rates before and after thermal solvent immersion annealing at 80 ˚C for 1 hour.

Condition	Strain Rate (mm/min)	Elastic Modulus (MPa)	Toughness (MJ/m^3)	Strain at Break (%)	Stress at Break (MPa)
Neat	5	267 ± 12	499 ± 19	1519 ± 33	49.4 ± 1.1
	10	268 ± 21	460 ± 11	1412 ± 20	47.6 ± 0.8
	20	283 ± 28	474 ± 20	1417 ± 37	50.8 ± 1.2
	40	371 ± 20	427 ±16	1334 ± 35	50.5 ± 1.1
Annealed	5	220 ± 20	372 ± 43	1287 ± 109	40.9 ± 2.7
	10	223 ± 17	395 ± 24	1331 ± 56	43.1 ± 1.5
	20	237 ± 21	404 ± 40	1315 ± 87	44.7 ± 2.6
	40	206 ± 14	362 ± 6	1226 ± 21	43.4 ± 0.5

Table S6. Average strain at break of simulated interfaces for neat and annealed samples (n=6).

Sample	Average Strain at Break (%)
Neat	40.8 ± 7.7
Anneal-L	52.0 ± 7.3
Anneal-M	69.7 ± 7.8
Anneal-F	102.9 ± 5.8

Table S7. Fracture mechanics of 70PP/30LDPE before and after thermal solvent immersion annealing

Bars (70PP/30LDPE)	b (mm)	λ_c	$W(\lambda_c)$ (MPa)	Γ (J/m^2)	Failure Mode
Neat	1.75	1.078	0.570	6980	Brittle
	1.25	1.057	0.319	3901	Brittle
	0.75	1.062	0.356	4359	Brittle
Annealed	1.75	1.254	2.700	33070	Ductile
	1.25	1.116	0.905	11088	Ductile
	0.75	1.066	0.365	4473	Brittle

Table S8. Mechanical properties of PP/PE blends before and after thermal solvent immersion annealing in xylenes.

Composition	Immersion Annealing T (^{0}C)	Elastic Modulus (MPa)	Toughness (MJ/m^3)	Strain at Break (%)	Stress at Break (MPa)
80PP/20LDPE	Neat	354 ± 9	11 ± 3	57 ± 15	17.6 ± 1.5
	80	246 ± 35	235 ± 24	1040 ± 14	30.4 ± 1.7
60PP/40LDPE	Neat	222 ± 11	4 ± 1	31 ± 3	16.0 ± 1.0
	80	197 ± 11	75 ± 6	556 ± 38	14.3 ± 0.5
85PP/15HDPE	Neat	458 ± 53	6 ± 1	28 ± 1	17.7 ± 1.9
	90	340 ± 23	203 ± 12	881 ± 32	27.1 ± 1.3
70PP/30HDPE	Neat	463 ± 44	3 ± 1	10 ± 2	25.0 ± 1.5
	90	328 ± 41	62 ± 5	392 ± 38	17.2 ± 0.3
60PP/40HDPE	Neat	297 ± 42	0.8 ± 0.1	7 ± 2	18.3 ± 0.2
	90	218 ± 13	9 ± 1	52 ± 5	12.9 ± 0.6
70PP/20LDPE/ 10HDPE	Neat	217	4	25	23.0
	80	209	136	660	23.8
50PP/30LLDPE/ 15LDPE/5HDPE	Neat	181	2	17	17.1
	60	176	77	527	15.6
30PP/30LLDPE/ 40LDPE	Neat	117	3	28	9.2
	70	143	61	629	10.4
70PP/30LLDPE	Neat	223 ± 9	126 ± 20	580 ± 83	26.6 ± 0.1
	70	260 ± 14	159 ± 12	836 ± 50	24.4 ± 0.7
30PP/70LLDPE	Neat	198 ± 1	102 ± 4	830 ± 6	15.1 ± 0.2
	70	141 ± 10	135 ± 8	1275 ± 38	14.8 ± 0.4

Table S9. Crystalline properties of PP/PE blends before and after thermal solvent immersion annealing in xylenes.

Composition	Immersion Annealing T (°C)	$T_{m,PP}$ (^{0}C)*	$T_{m,PE}$ (^{0}C)*	$X_{c,PP}$*	$X_{c,PE}$*
80PP/20LDPE	Neat	157.3	105.6	0.38	0.11
	80	160.0	98.5	0.30	0.11
60PP/40LDPE	Neat	157.2	104.6	0.31	0.17
	80	161.2	100.9	0.43	0.10
85PP/15HDPE	Neat	160.3	130.4	0.33	0.57
	90	160.2	130.7	0.32	0.72
70PP/30HDPE	Neat	159.7	132.2	0.33	0.57
	90	160.0	131.6	0.32	0.59
70PP/30LLDPE	Neat	158.9	121.3	0.42	0.09
	70	156.3	120.6	0.40	0.06
30PP/70LLDPE	Neat	158.0	120.3	0.30	0.14
	70	160.6	122.5	0.24	0.19

*Measurements collected from first heats of DSC scans

Table S10. Mechanical properties of state-of-the-art compatibilized blends of PP/PE.

Blend Composition	Compatibilizer Loading (wt.%)	Strain at Break (%)	Modulus (MPa)	Supplemental Reference
30PP/70HDPE	0.5	550	*	6
30PP/70HDPE	1.0	535	*	6
30PP/70HDPE	5.0	600	*	10
30PP/70HDPE	1.0	550	*	11
30PP/70HDPE	0.5	220	*	11
30PP/70HDPE	3.0	800	*	11
30PP/70HDPE	5.0	810	*	11
70PP/30HDPE	0.5	410	*	11
70PP/30HDPE	1.0	550	*	11
70PP/30HDPE	3.0	800	*	11
70PP/30HDPE	5.0	790	*	11
90PP/10HDPE	5.0	726	2200	12
80PP/20HDPE	5.0	656	2200	12
30PP/70HDPE	5.0	950	*	13
30PP/70HDPE	5.0	100	*	13
30PP/70HDPE	5.0	57	*	13
30PP/70HDPE	5.0	850	*	13

30PP/70HDPE	5.0	990	*	[13]
30PP/70HDPE	5.0	210	*	[13]
30PP/70HDPE	5.0	910	*	[13]
30PP/70HDPE	5.0	620	*	[13]
30PP/70HDPE	5.0	860	*	[13]
30PP/70HDPE	5.0	910	*	[13]
30PP/70HDPE	1.0	200	*	[13]
30PP/70HDPE	5.0	150	*	[13]
30PP/70HDPE	1.0	600	*	[13]
30PP/70HDPE	1.0	190	*	[13]
30PP/70HDPE	1.0	850	*	[13]
70PP/30PE**	3.0	464	*	[14]
70PP/30HDPE	2.5	360	*	[15]
70PP/30HDPE	5.0	690	*	[15]
40PP/60PE**	0.2	766	300	[16]
40PP/60PE**	10.0	719	200	[16]
70PP/30HDPE	10.0	460	900	[17]
50PP/50LLDPE	5.0	700	685	[18]
30PP/70HDPE	5.0	100	900	[18]
70PP/30HDPE	5.0	433	863	[19]
30PP/70HDPE	5.0	67	832	[19]
20PP/80PE**	3.0	125	1036	[20]
80PP/20LDPE	5.0	36	1780	[21]
60PP/40LDPE	5.0	50	1360	[21]
40PP/60LDPE	5.0	47	1020	[21]
20PP/80LDPE	5.0	49	890	[21]
75PP/25LDPE	10.0	400	1100	[22]
70PP/30HDPE	5.0	422	1138	[23]
70PP/30HDPE	5.0	193	1144	[23]
70PP/30HDPE	5.0	179	1206	[23]
70PP/30HDPE	5.0	259	1269	[23]
70PP/30HDPE	1.0	145	1440	[24]
30PP/70HDPE	1.0	151	1390	[24]

*Value not reported
**PE type not specified

SUPPLEMENTAL REFERENCES

(1) Zhang, Y.; Zhang, W. Course-Grained Simulations of Crystallization in Phase-Separated Polymer Blends with Block Copolymer Compatibilizers. *Macromolecules* **2025**.

(2) Mencik, Z. Crystal Structure of Isotactic Polypropylene. *Journal of Macromolecular Science, Part B* **1972**, *6* (1), 101–115. https://doi.org/10.1080/00222347208224792.

(3) Alsaygh, A. A.; Al-hamidi, J.; Alsewailem, F. D.; Al-Najjar, I. M.; Kuznetsov, V. L. Characterization of Polyethylene Synthesized by Zirconium Single Site Catalysts. *Appl. Petrochem. Res.* **2014**, *4* (1), 79–84. https://doi.org/10.1007/s13203-014-0053-2.

(4) Jeon, H. S.; Lee, J. H.; Balsara, N. P. Predictions of the Thermodynamic Properties of Multicomponent Polyolefin Blends from Measurements on Two-Component Systems. *Macromolecules* **1998**, *31* (10), 3328–3339. https://doi.org/10.1021/ma9709718.

(5) Hiemenz, P. C.; Lodge, T. P. *Polymer Chemistry*; CRC Press, 2007. https://doi.org/10.1201/9781420018271.

(6) Xu, J.; Eagan, J. M.; Kim, S. S.; Pan, S.; Lee, B.; Klimovica, K.; Jin, K.; Lin, T. W.; Howard, M. J.; Ellison, C. J.; Lapointe, A. M.; Coates, G. W.; Bates, F. S. Compatibilization of Isotactic Polypropylene (IPP) and High-Density Polyethylene (HDPE) with IPP-PE Multiblock Copolymers. *Macromolecules* **2018**, *51* (21), 8585–8596. https://doi.org/10.1021/acs.macromol.8b01907.

(7) Kröger, M.; Dietz, J. D.; Hoy, R. S.; Luap, C. The Z1+ Package: Shortest Multiple Disconnected Path for the Analysis of Entanglements in Macromolecular Systems. *Comput. Phys. Commun.* **2023**, *283*, 108567. https://doi.org/10.1016/j.cpc.2022.108567.

(8) Hu, W.-G.; Schmidt-Rohr, K. Polymer Ultradrawability: The Crucial Role of α-Relaxation Chain Mobility in the Crystallites. *Acta Polymerica* **1999**, *50* (8), 271–285. https://doi.org/10.1002/(SICI)1521-4044(19990801)50:8<271::AID-APOL271>3.0.CO;2-Y.

(9) Zhang, Y.; Zhang, W. Effects of Block Copolymer Compatibilizers and Interfacial Entanglements on Strengthening Immiscible Glassy Polymer Blends. *Macromolecules* **2025**, *58* (5), 2484–2493. https://doi.org/10.1021/acs.macromol.4c02848.

(10) Eagan, J. M.; Xu, J.; Di Girolamo, R.; Thurber, C. M.; Macosko, C. W.; La Pointe, A. M.; Bates, F. S.; Coates, G. W. Combining Polyethylene and Polypropylene: Enhanced Performance with PE/IPP Multiblock Polymers. *Science (1979).* **2017**, *355* (6327), 814–816. https://doi.org/10.1126/science.aah5744.

(11) Shen, L.; Gorbea, G. D.; Danielson, E.; Cui, S.; Ellison, C. J.; Bates, F. S. Threading-the-Needle: Compatibilization of HDPE/IPP Blends with Butadiene-Derived Polyolefin Block Copolymers. *Proceedings of the National Academy of Sciences* **2023**, *120* (34), e2301352120. https://doi.org/10.1073/pnas.2301352120.

(12) Kruszynski, J.; Nowicka, W.; Rozanski, A.; Liu, Y.; Parisi, D.; Yang, L.; Pasha, F. A.; Bouyahyi, M.; Jasinska-Walc, L.; Duchateau, R. PP/HDPE Blends Compatibilized by a Polyester: An Unconventional Concept to Valuable Products. *Sci. Adv.* **2024**, *10* (21), eado1944. https://doi.org/10.1126/sciadv.ado1944.

(13) Klimovica, K.; Pan, S.; Lin, T.-W.; Peng, X.; Ellison, C. J.; LaPointe, A. M.; Bates, F. S.; Coates, G. W. Compatibilization of IPP/HDPE Blends with PE-g-IPP Graft Copolymers. *ACS Macro Lett.* **2020**, *9* (8), 1161–1166. https://doi.org/10.1021/acsmacrolett.0c00339.

(14) Coba-Daza, S.; Carmeli, E.; Otaegi, I.; Aranburu, N.; Guerrica-Echevarria, G.; Kahlen, S.; Cavallo, D.; Tranchida, D.; Müller, A. J. Effect of Compatibilizer Addition on the Surface Nucleation of Dispersed Polyethylene Droplets in a Self-Nucleated Polypropylene Matrix. *Polymer (Guildf).* **2022**, *263*, 125511. https://doi.org/10.1016/j.polymer.2022.125511.

(15) Kränzlein, M.; Cui, S.; Hu, J.; LaPointe, A. M.; Fors, B. P.; Coates, G. W. One-Step Radical-Induced Synthesis of Graft Copolymers for Effective Compatibilization of Polyethylene and Polypropylene. *J. Am. Chem. Soc.* **2025**, *147* (22), 19052–19060. https://doi.org/10.1021/jacs.5c03869.

(16) Zou, S.; Li, D.; Wang, W.; Li, S.; Long, S.; Huang, Y.; Li, X. Compatibilization of Polyethylene and Polypropylene via Dynamic Cross-Linking. *Macromolecules* **2025**, *58* (21), 11883–11891. https://doi.org/10.1021/acs.macromol.5c02898.

(17) Lin, Y.; Yakovleva, V.; Chen, H.; Hiltner, A.; Baer, E. Comparison of Olefin Copolymers as Compatibilizers for Polypropylene and High-density Polyethylene. *J. Appl. Polym. Sci.* **2009**, *113* (3), 1945–1952. https://doi.org/10.1002/app.30190.

(18) Banerjee, A.; Kafle, N.; Romano, W. G.; Pandya, H.; Horiuchi, S.; Srivastava, A.; Khabaz, F.; Foster, M. D.; Miyoshi, T.; Eagan, J. M. Segmented Multiblock Polyolefin Compatibilizers from Non-Living Metathesis Chain-Shuffling. *Nat. Commun.* **2025**, *16* (1), 10515. https://doi.org/10.1038/s41467-025-65525-1.

(19) Tal, A.; Naveh, N. Compatibilization of Post-Consumer Recycled Polypropylene/Polyethylene Binary Blends. *Polym. Adv. Technol.* **2024**, *35* (11). https://doi.org/10.1002/pat.6645.

(20) Graziano, A.; Titton Dias, O. A.; Sena Maia, B.; Li, J. Enhancing the Mechanical, Morphological, and Rheological Behavior of Polyethylene/Polypropylene Blends with Maleic Anhydride-grafted Polyethylene. *Polym. Eng. Sci.* **2021**, *61* (10), 2487–2495. https://doi.org/10.1002/pen.25775.

(21) Penava, N. V.; Rek, V.; Houra, I. F. Effect of EPDM as a Compatibilizer on Mechanical Properties and Morphology of PP/LDPE Blends. *Journal of Elastomers & Plastics* **2013**, *45* (4), 391–403. https://doi.org/10.1177/0095244312457162.

(22) Su, B.; Zhou, Y.-G.; Wu, H.-H. Influence of Mechanical Properties of Polypropylene/Low-Density Polyethylene Nanocomposites. *Nanomaterials and Nanotechnology* **2017**, *7*, 184798041771592. https://doi.org/10.1177/1847980417715929.

(23) Wolff, P.; Dickert, A.; Kretschmer, W. P.; Kempe, R. IPP/PE Multiblock Copolymers for Plastic Blend Recycling Synthesized by Coordinative Chain Transfer Polymerization. *Macromolecules* **2022**, *55* (15), 6435–6442. https://doi.org/10.1021/acs.macromol.2c00709.

(24) Gorbea, G. D.; Shen, L.; Flanigan, K.; Ellison, C. J.; Bates, F. S. Enhancing Toughness of Post-Consumer Recycled Polyolefins with Polybutadiene-Derived Block Copolymers. *ACS Appl. Polym. Mater.* **2024**, *6* (20), 12691–12699. https://doi.org/10.1021/acsapm.4c02270.
